# Hydrophobic Lightweight Cement with Thermal Shock Resistance and Thermal Insulating Properties for Energy-Storage Geothermal Well Systems

**DOI:** 10.3390/ma14216679

**Published:** 2021-11-05

**Authors:** Toshifumi Sugama, Tatiana Pyatina

**Affiliations:** Brookhaven National Laboratory, Upton, NY 11973-5000, USA; sugama@bnl.gov

**Keywords:** lightweight cement, thermally insulating cement, energy storage wells, geothermal cement, thermal-shock-resistant cement, hydrophobic cement

## Abstract

This study assessed the possibility of using polymethylhydrosiloxane (PMHS)-treated fly ash cenospheres (FCS) for formulating a thermally insulating and thermal shock (TS)-resistant cementitious blend with calcium aluminate cement. To prevent FCS degradation in an alkaline cement environment at high temperatures, the cenospheres were pre-treated with sodium metasilicate to form silanol and aluminol groups on their surface. These groups participated in a dehydrogenation reaction with the functional ≡Si–H groups within PMHS with the formation of siloxane oxygen-linked M-FCS (M: Al or Si). At high hydrothermal temperatures of 175 and 250 °C, some Si–O–Si and SiCH_3_ bonds ruptured, causing depolymerization of the polymer at the FCS surface and hydroxylation of the raptured sites with the formation of silanol groups. Repolymerization through self-condensation between the silanol groups followed, resulting in the transformation of siloxane to low crosslinked silicon-like polymer as a repolymerization-induced product (RIP) without carbon. The RIP provided adequate protection of FCS from pozzolanic reactions (PR), which was confirmed by the decline in zeolites as the products of PR of FCS. Cements with PMHS-treated FCS withstood both hydrothermal and thermal temperature of 250 °C in TS tests, and they also showed improved compressive strength, toughness, and water repellency as well as decreased thermal conductivity. The lubricating properties of PMHS increased the fluidity of lightweight slurries.

## 1. Introduction

The use of generating or storage geothermal systems for stabilizing the electric grid, increasing its flexibility, and providing energy on demand presents a possibility of flexible and renewable energy generation and storage. The geothermal environments of storage reservoirs can range from rock and sand formations to hot confined aquifers over the bedrock heated by magma. For a robust long-term performance, the geothermal reservoir must be able to withstand repeated thermal stresses during the injection and production of heated fluids. Since the most economical heat-recovery systems require high-temperature carrier fluids, the thermal shocks may be significant. For example, in case of hybrid solar–geothermal energy production and storage, the steam temperature may exceed 300 °C. Cement is used for well construction as a sheath between metallic pipes (will be referred to as “casing” in the paper) and underground formation zones to provide zonal isolation, prevent casing corrosion, and support the well structure. Cement sheath failure to fulfill any of its tasks may result in catastrophic events where the well would be abandoned, and a new well would have to be constructed. Although generally, it is difficult to attribute the failure of a well to a single factor, cement failure was concluded to cause the failure in several geothermal fields [1,2]. Oil field well integrity solutions cannot be easily adopted for geothermal wells because of the high operating temperatures, aggressive environments, and repeated thermal shock (TS) conditions.

In storage wells, casing and cement sheath undergo TS during the injection–production cycles, resulting in repeated thermal stresses by thermal expansion of the casing (hot fluid passing through the casing, compressive stress on cement sheath) or casing contraction (cold fluid passing through the casing, tensile stress on cement sheath) causing, in the first case, the development of microcracks in the cement sheath and, in the second case, the development of a micro-annulus between the sheath and the casing [3].

To deal with the TS concern in geothermal and energy-storage wells, the alkali (sodium metasilicate, SMS)-activated calcium aluminate cement (CAC)/Class F fly ash (fly ash F, FAF) blend cements were evaluated to develop TS-resistant cement (TSRC) [4]. The integrity of cement matrix and the bond durability at interfaces between cement sheath and carbon steel casing under repeated hot–cool thermal stress cycles were evaluated. The matrix integrity was tested in a five-cycle 600 °C heating → cold water quenching TS test on 200 °C-autoclaved samples. The post-TS test analysis revealed the occurrence of three crystalline phase-transformations: boehmite from CAC → γ-Al_2_O_3_, katoite from CAC → calcite, and hydroxysodalite zeolite as an alkali-activated hydrothermal reaction product between CAC and FAF → carbonated sodalite. Among those, the hydroxysodalite → carbonated sodalite transformation played a pivotal role in densifying the cementitious structure, and in sustaining the material’s original compressive strength developed during the autoclaving; additionally, it improved the resistance of the refractory CAC to thermal shock.

For the adherence of cement sheath to carbon steel casing, five cycles of TS test (one cycle: 350 °C–24 h heating followed by passing cold water through the casing for 10 min) were performed for the cement-sheathed casing samples prepared by autoclaving at 300 °C [5,6]. As a result, the major factor governing the TS cement-casing bond durability was the great adhesive ductility of TSRC. The contributors to such ductile adhesive behavior included amorphous NaO_2_-CaO-Al_2_O_3_-SiO_2_-H_2_O, NaO_2_-Al_2_O_3_-SiO_2_-H_2_O as alkali-activated hydrothermal CAC/FAF reaction products, and silica gel phases as major hydration products in conjunction with three crystalline phases, analcime (CAC/FAF reaction product) and cancrinite (carbonated CAC/FAF reaction product), and boehmite as minor ones, which were formed in the critical cement/casing interfacial region. Particularly, these amorphous phases more likely improved the bond durability (rather than crystalline phases) because of their ductile nature.

In addition to TS, another important concern in energy storage and geothermal energy-generation wells is heat losses. The rate of heat loss will depend on various parameters including well depth and temperature. As an example, in the case of a 3.2 km deep well, the working fluid of 93 °C at the bottom of the well will have a temperature of 33 °C when the fluid will reach the well head, which corresponds to a 64.5% temperature decrease. In contrast, if an insulated cementitious material with low thermal conductivity of 0.45 W/mK is deployed between metal casing and the formation in this well, the data obtained from the modeling work of insulated geothermal well structure reveal that the heat loss rate to the rock formation is only 16.1% [7]. Importantly, the insulating well cement sheath must have low thermal conductivity under water-saturated conditions of an underground well and not under dry conditions. Such insulating cement technology would be applicable to energy-recovery wells or any storage well, including subsurface compressed air energy storage, subsurface pumped hydroelectric storage, hot-water thermal energy storage, and aquifer thermal energy storage. Since the use of high-temperature geothermal wells with the hydrothermal temperature of 250 °C is a new approach to energy storage, to the best of the authors’ knowledge, the formulation and evaluation of thermally insulating hydrothermal cement possessing 250 °C ↔ 25 °C cycling TS resistance has not been reported.

The thermal conductivity (TC) of the subsurface rock- and clay-based formations in the top 300 m was reported to be in the range of 2.30–3.86 W/mK for most Precambrian granites and sandstones in the geothermal heat exchange systems in Wisconsin [8]. Exceptionally, Barron quartzite and St. Lawrence dolomite in this rock formation exhibited a very high TC value of 6.71 and 4.67 W/mK, respectively [9]. The TC values of all common clays, such as Ca-smectite, Na-smectite, illite, and palygorskite were similar, ranging from 0.69 to 0.80 W/mK at a dry density of 1.6 g/cm^3^. The TC depended primarily on the density and water content of clays; namely, the increase in density to 1.8 g/cm^3^ from dry density of 1.2 g/cm^3^ by absorption of 17% of water resulted in the rise of TC value to ≈1.32 W/mK from ≈0.63 W/mK for a dry clay [10].

The TC of materials and chemical components present in a geothermal well structure decreases in the following order: steel casing (≈58 W/mK) >> rock formation (≈1.8 W/mK) > cement sheath (≈0.93 W/mK) > free water (≈0.61 W/mK) >> air (≈0.026 W/mK). The air possesses the lowest TC, which is nearly 25 times lower than that of water, while TC of the rock formation is nearly two and three times higher than that of cement and water, respectively, and clays have 1.4 times higher TC values. On the other hand, the hydrated cement sheath is composed primarily of mineral-based crystalline/amorphous compounds with water molecules intercalated in these compounds along with some free water. Since the TC of water in hydrated cement is more than 50% higher than that of free water [11], the mineral-based hydrated cement exhibits higher TC than free water. Thus, there are three possible strategies to reduce the cement sheath’s TC and improve its thermal insulation property. The first one is to incorporate a large volume of gas into the cement; the second is to reduce the volume of the hydrated cement matrix; and the third is to minimize the free water content through decreased water absorption by the cement sheath and slurry water content. The insulating TSRC must also possess adequate mechanical properties.

Fly ash cenospheres (FCS), the lightweight hollow aggregates made of a dense pozzolanic shell that incapsulates CO_2_ (major) and N_2_ (minor) gas phases [12], can be used to improve thermally insulating properties of TSRC at geothermal temperature of 250 °C. Since both FCS and FAF, used in TSRC, are by-products of coal combustion power plants, the chemical constituents of FCS’s shells are similar to those of FAF; namely, they are pozzolans containing silica and aluminum silicate mineral mullite (3Al_2_O_3_·2SiO_2_) [13]. More importantly, both materials are compatible with the TSRC formulation. The diameter of spherical FCS is in the range between 5 and 500 µm, and the shell thickness is between 2 and 30 µm [14,15,16]. The TC of FCS is in the range of 0.13 to 0.38 W/mK, and the bulk density of the material is 0.3–0.5 g/cm^3^ [17,18].

Several investigators studied FCS’s use as a thermally insulating material for high-strength lightweight concretes in the building and construction industry [19,20,21,22,23]. For instance, Ordinary Portland Cement (OPC)/FCS blends with the density of 1.09 and 1.41 g/cm^3^ reached compressive strength of 5.0 and 30.1 MPa and TC values (dry conditions) of 0.36 and 0.45 W/mK respectively after 28 days of hydration [20]. A geopolymer composite system of fly ash/FCS/metakaolin [23] showed compressive strength of 36.5 MPa and TC of 0.173 W/mK for samples with dry density of 0.82 g/cm^3^.

One important concern of using FCS as part of cementitious systems is the pozzolanic reactivity of their shells in alkaline environments containing OH^−^, Na^+^, Ca^2+^, and Al^3+^ ions. Pozzolanic reactions of the FCS’s shell can cause the volumetric expansion-induced failure of cements. Although such reactivity at low temperatures of 28–30 °C was minimal for OPC/FCS blends, an elevated temperature of 80 °C led to the acceleration of pozzolanic reactions with alkalis liberated from OPC to form aluminum-substituted tobermorite [Ca_5_Si_5_Al(OH)O_17_5H_2_O] identified as the main reaction product [24]. Likewise, in the alkali-activated TSRC system, the FCS shells may be susceptible to pozzolanic reactions with SMS used as an alkali activator of FAF. In solutions, SMS forms three types of ions, Na^+^, OH^−^, and metasilicate anion, SiO_3_^2−^. The pozzolanic reactions of FCS shells with alkali ions, OH^−^ and Na^+^, may promote shells’ degradation after a certain period of exposure in hydrothermal environment at high temperature ≥250 °C. If so, the degradation may cause undesirable consequences, including a loss of thermal insulating capability by increased water absorption along with a loss of gaseous phases from the FCS, and a reduction of the mechanical strength of the cement sheath due to pozzolanic reactions-induced swelling and expansion of cement matrix.

To avoid FCS degradation in high-temperature alkaline environments, their surfaces may be treated with pozzolanic reactions-inhibiting agents. Polymethylhydrosiloxane (PMHS) can be a suitable surface-treating chemical against the pozzolanic reactions of FCS. As already documented [25,26,27,28], PMHS is a hydrophobic, reducing agent that has siloxane backbone chains, (–Si–O–Si-O–)_n_, with two types of functional groups: proton-donor Si–H and hydrophobic Si–CH_3_, representing the –[–Si(CH_3_)_2_–O–]_m_-[–HSi(CH_3_)–O–]_n_– structure. The functional Si–H reacts with hydroxyl (–OH) groups on any organic and inorganic substrates. Hydrogen is released during this interaction in the following dehydrogenation reaction: 2Si–H + 2OH-linked substrates → 2Si–O-substates + 2H_2 (gas)_↑. Then, oxygen bridges form between functional proton-depleted polymethylsiloxane (PMS) and substrates, resulting in the substrate surface coverage with hydrophobic –Si(CH_3_) groups attached to PMS chains. The surface of the substrate becomes water-repellent and hydrophobic. Most importantly, hydrophobic cement concrete provides good thermal insulating properties [29].

Hence, the above information strongly suggested that if FCS shell surfaces can be functionalized with –OH groups, PMHS is applicable as pozzolanic reaction-inhibiting surface treatment additive. A SMS alkali activator, which is a part of the TSRC formulation, may be used to incorporate –OH groups into the surface of FCS shell. In fact, the pozzolanic reactions of zeolite, silica, and clay minerals in alkali environments were shown to create Al(OH)_4_- and SiO(OH)_3_-anionic reactants on the mineral surfaces [30,31,32,33].

Regarding the thermal stability of PMHS, thermogravimetric study showed that the continuous weight loss of this chemical occurred at temperatures, ranging from 160 to 340 °C. At over 340 °C, a considerable loss of weight was due to thermal decomposition of backbone Si–O–Si linkages [34,35]. Thus, PMHS appears to possess a good thermal stability, but there is no information available for its hydrothermal stability in alkali environments at elevated temperatures.

Based upon the above information, the objective of this study was to formulate and evaluate lightweight, insulating cement composite with FCS, which is stable at hydrothermal temperature of 250 °C and able to withstand three thermal shock cycles (one cycle: 24 h 250 °C heat → cold water quenching). To achieve low thermal conductivity under the water-saturated conditions, the composite would include FCS treated with PMHS to avoid pozzolanic reactions. Additionally, the composite would need to have mechanical properties acceptable for high-temperature geothermal wells, including compressive strength of no less than 3.4 MPa.

## 2. Materials and Methods

### 2.1. Starting Materials

**Figure 1:**
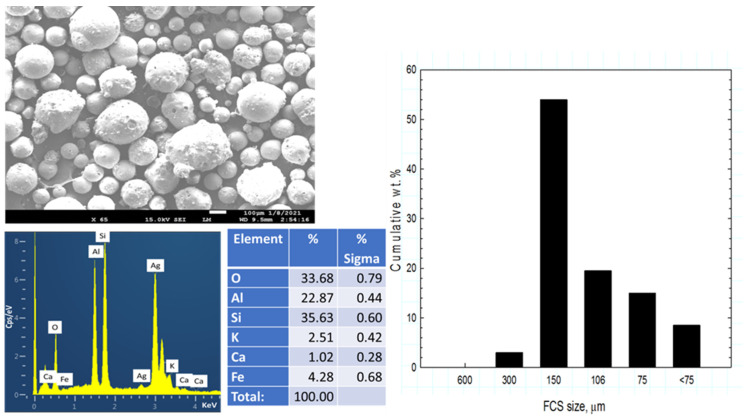
Photomicrograph, elemental analysis, and cumulative size distribution of tested FCS.

Secar #80 calcium aluminate cement (CAC) was supplied by Imerys Inc (Greenevile, TN, USA). The X-ray powder diffraction (XRD) data showed that the crystalline compounds of CAC were three principal phases: calcium monoaluminate (CaO·Al_2_O_3_, CA), calcium dialuminate (CaO·2Al_2_O_3_, CA_2_), and corundum (α-Al_2_O_3_). CenoStar Corp.( Newburyport, MA, USA) provided fly ash cenospheres (FCS) under the trade name “CenoStar ES500.” The “as-received” FCS had bulk density of 0.32–0.45 g/cm^3^ and thermal conductivity of 0.1–0.2 W/mK. Figure 1 shows Scanning Electron Microscope (SEM) image and Energy-Dispersive X-ray (EDX) elemental analysis along with the particle size distribution for the FCS. EDX analysis gave the following elemental composition: 33.7% O, 22.9% Al, and 35.6% Si as the major elements, and 2.5% K, 1.0% Ca, and 4.3% Fe as the minor ones. Ag was used as a coating material to avoid surface charging. The cumulative size distribution of FCS was 3 wt.% 300 µm, 54 wt.% 150 µm, 19.5 wt.% 106 µm, 15 wt.% 75 µm, and 8.5 wt.% < 74 µm. Table 1 shows the oxide compositions of these starting materials.

XRD analysis showed two crystalline phases in the composition of FCS, mullite (ICDD#04-016-1586, Al_2.22_Si_0.78_ O_4.89_) and silica (#04-008-8437, SiO_2_). Class F fly ash was obtained from Boral Material Technologies (Antonio, TX, USA), and its crystalline phase composition was similar to that of FCS. A 93% sodium metasilicate (SMS, Na_2_SiO_3_) powder with a particle size of 0.23 to 0.85 mm under the trade name “MetsoBeads 2048” was supplied by PQ Corporation (Avenel, NJ, USA) and had a 50.5/46.6 Na_2_O/SiO_2_ molecular weight ratio. Polymethylhydrosiloxane (PMHS), (CH_3_)_3_SiO-[(CH_3_)HSiO]_n_-Si(CH_3_)_3_, with the density of 15–40 cP at 20 °C, was obtained from Sigma-Aldrich (St. Louis, MO, USA).

### 2.2. Samples Preparation

#### 2.2.1. Optimization of SMS Content

**Figure 2:**
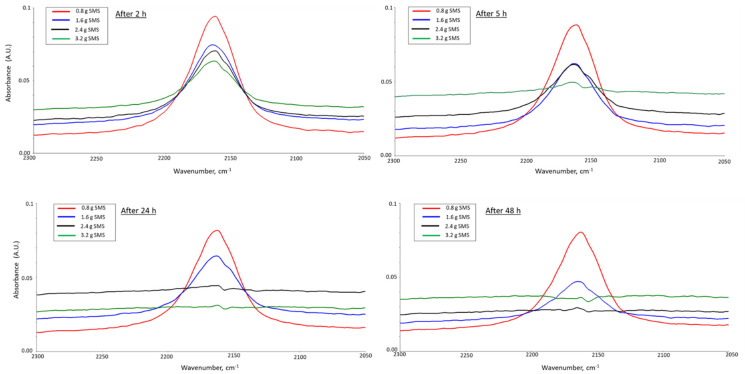
Comparison of spectral features of Si–H band at 2163 cm^−1^ after 2, 5, 24, and 48 h for PMHS-treated FCS samples made with various amounts of SMS catalyst.

**Figure 3:**
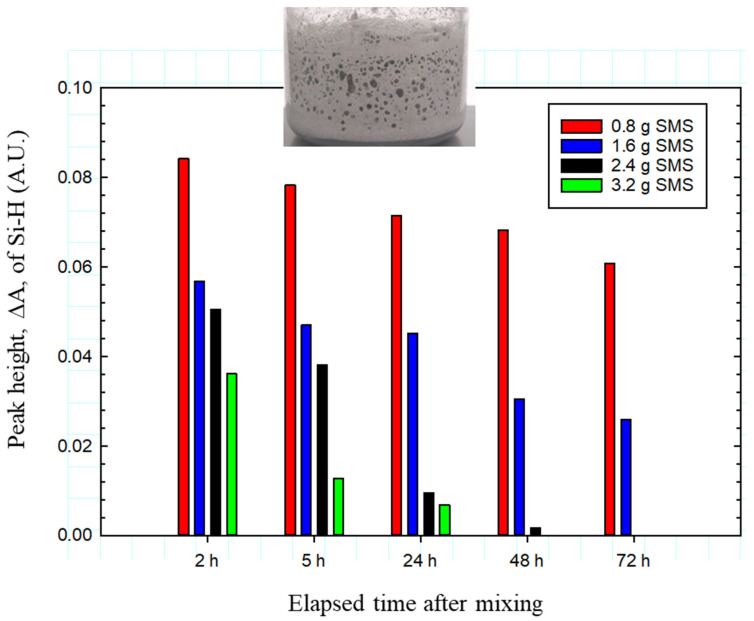
Changes in peak height, ∆A, of Si–H band as a function of elapsed time from the start of the reaction for PMHS/FCS blends in the presence of SMS.

To establish the treatment procedure of FCS with PMHS in the presence of SMS, firstly, the appropriate concentration of the SMS and the treatment time were determined. Different amounts of SMS (0.8, 1.6, 2.4, and 3.2 g) were dissolved in 15 g of water. To each of the SMS solutions, 1.2 g of PMHS was added and mixed for 30 min with a magnetic stirrer. Thereafter, 16 g of FCS was incorporated into the PMHS/SMS blend and mixed by hand for 30 s to prepare paste-like samples. In the following two days, the blends were hand-mixed 4 times a day with a 2 h interval. The extent of the dehydrogenation reaction of Si–H functional groups within PMHS with the OH groups on the FCS surfaces was monitored by Attenuated Total Reflectance-Fourier Transform Infrared Spectroscopy (ATR-FTIR). There are two IR absorption bands for Si-H bond [36,37,38]: one is stretching vibration (*ν_Si–H_*) at ≈2160 cm^−1^; the other is bending vibration (*δ_Si–H_*) at 828 cm^−1^. We monitored the decrease in the peak height, ∆A, of the band at 2163 cm^−1^ as a function of SMS amount as well as the elapsed reaction time between two hours after the sample preparation and up to 3 days. The decreased ∆A value can be accounted for by reacting Si–H groups with OH groups and NaOH. Figure 2 depicts the changes in spectral features for the different amounts of SMS after the elapsed times of 2, 5, 24, and 48 h. Correspondingly, Figure 3 shows the summarized results of ∆A values as a function of time elapsed after sample mixing. For all samples, ∆A decreased as the reaction time increased. The rate of ∆A decrease depended primarily on the SMS content. As expected, higher SMS concentrations accelerated the dehydrogenation reaction of Si–H. The reaction was accompanied by the release of H_2_, which resulted in the blend foaming (see the photograph in Figure 3).

In fact, after 2 h of the reaction, the ∆A value of 0.036 was 2.3 times lower for the slurry with the highest SMS content of 3.2 g than that for the slurry with the lowest SMS content of 0.8 g. After 24 h of the reaction, the ∆A value decreased to 0.007 for the slurry with 3.2 g SMS. This was nearly an order of magnitude lower than for the slurry with 0.8 g SMS. With the highest SMS concentration, the band at 2163 cm^−1^ disappeared completely, strongly suggesting that most of the Si–H groups reacted with the release of hydrogen. With 2.4 g SMS, the reaction of Si–H seems to be completed after 72 h, according to the ATR-FTIR results. These data suggest that SMS plays an important role in the PMHS dehydrogenation reactions.

#### 2.2.2. Cement Formulation and Sample Preparation Sequence

Based on the described above determination of the SMS concentration necessary for the PMHS/FCS treatment, the surface treatment of FCS by PMHS was accomplished in the following three-step process (the percentage of materials is given by the total weight of the blend: 60 wt.%CAC/40 wt.%FCS): Step (1) preparation of SMS solution (6% SMS in 38% water) → (2) PMHS blending with SMS solution → (3) addition of 40% FCS to PMSH-blended SMS solution. Thereafter, the PMHS-treated FCS paste was incorporated into 60% CAC as a cementitious binder with additional water in the range between 10 and 19% to prepare the lightweight cement slurry. The cement samples were prepared in the following four steps: (1) pouring cement slurry into test molds, followed by 24 h curing at room temperature → (2) removal of cured cement from the molds → (3) curing the samples in a 99 ± 1% relative humidity for 24 h at 85 °C → (4) autoclaving for 24 h at 175° or 250 °C.

For comparison, regular density TSRC consisting of 60 wt.% CAC/40 wt.% FAF and 6% SMS by total weight of CAC and FAF blend was used as a control. The water-to-cement ratio (where cement is a blend of CAC and FAF) (W/C) was 0.58. The curing of this cement was done in the same manner as the lightweight cement.

To identify the reaction products formed in the preparation of PMHS-modified FCS in SMS solution at 85°, 175°, and 250 °C, the FCS/SMS, PMHS/SMS, and PMHS/FCS/SMS systems were prepared at the same mass ratios as for the formulation described above. The systems were exposed to steam at 85 °C for 24 h; then, they were dried for 24 h in an oven at 100 °C for ATR-FTIR and XRD analyses. Next, these dried samples were autoclaved at 175° and 250 °C for 24 h, dried, and analyzed once more.

### 2.3. Measurements

To evaluate slurry flowability, slump was measured by using a polyethylene cone with the top hole of 20 mm diam., bottom hole of 45 mm diam., and 40 mm height. The cone was placed on a flat carbon steel plate, filled with cement slurry, and slowly lifted, allowing the slurry to spread. The slurry slump was measured 20 s later.

The cement resistance to TS was evaluated in a three-cycle TS test (one cycle: 250 °C heating for 24 h, followed by quenching in water at 25 °C).

The free water content was determined in the following manner. Before TS tests, the excess water was wiped of the surface of autoclaved samples with a paper towel. Then, the samples were placed in a vacuum oven for 3 days at 60 °C to eliminate removable free water. The free water content, wt.%, was computed by (W_wet_ − W_dry_)/W_wet_ × 100, where W_wet_ is the weight of a wiped water-saturated sample after the autoclave and W_dry_ is the weight of the dried sample. After the TS tests, the samples were immersed in water at ambient temperature for at least 4 days until the weight of the samples became constant. Afterward, the free water was calculated in the same manner as that of the samples before the TS.

To determine the compressive strength, Young’s modulus, and compressive toughness, the composite samples were prepared in the following sequence. The hand-mixed slurries were poured in cylindrical molds (20 mm diam. and 40 mm height) and left to harden for 1 day at room temperature; thereafter, the hardened cements were removed from the molds and placed in a 99 ± 1% relative humidity (R.H.) for 1 day at 85 °C; finally, the samples were autoclaved in non-stirred Parr Reactor 4622 (Hillsboro, OR, USA) for 1 day at 175° and 250 °C. An Electromechanical Instorn System Model 5967 (Norwood, MA, USA) was used to obtain all mechanical properties. The ultimate compressive strength and Young’s modulus were determined for unconfined samples. The compressive toughness was computed from the area under the compressive stress–strain curve.

Thermal conductivity (TC) was measured with a Quick Thermal Conductivity Meter, OTM-500, Kyoto Electronic, Kyoto, Japan) on rectangular prism samples (60 mm wide, 120 mm long, and 20 mm thick). The samples for TC measurements were prepared in the same manner as described above. Contact angle measurement for water droplets on 7-day-air-dried cement surfaces was done by Model CAA 3, Imass Inc.(Marshfield, MA, USA), using rectangular prism samples (15 mm wide, 75 mm long, 2 mm thick). The measurements were performed at ambient temperature. The contact angle was determined 20 s after placing a water droplet on cement samples. XRD (40 kV, 40 mA copper anode X-ray tube) and ATR-FTIR were used to identify amorphous and crystalline phase compositions and phase transitions of tested samples. The PDF-4/Minerals 2021 database of International Center for Diffraction Data (ICDD) was used for analyses of XRD patterns.

JEOL 7600F SEM (Pleasanton, CA, USA) image analysis coupled with EDX elemental composition survey of typical areas of fractured cement surfaces were done to determine two physicochemical factors: microstructural development at FCS and cement matrix interfaces and pozzolanic reaction products.

## 3. Results and Discussion

### 3.1. Identification of the Reaction Products and Mechanisms of Reactions between SMS, FCS, and PMHS at Hydrothermal Temperatures of 85, 175, and 250 °C

To identify the reaction products and establish mechanisms of the reactions at three different hydrothermal temperatures, we studied the following systems: FCS/SMS, PMHS/SMS, and PMHS/FCS/SMS.

#### 3.1.1. Reactions between FCS and SMS

**Figure 4:**
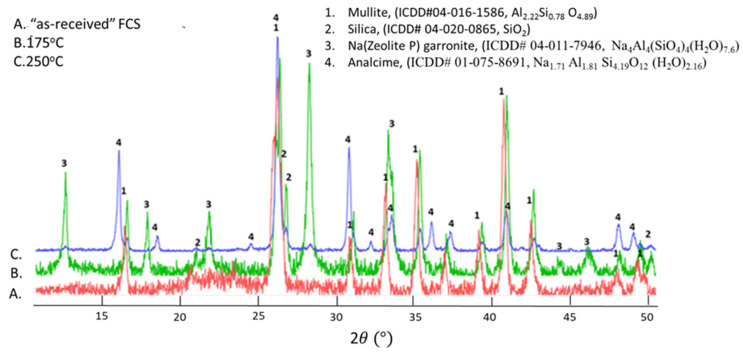
XRD patterns of “as-received” FCS, 175°- and 250°-autoclaved FCS/SMS blends.

Figure 4 shows XRD patterns of crystalline reaction products of FCS autoclaved with SMS at 175 and 250 °C as well as the XRD pattern of the FCS itself. As expected, the two major crystalline compounds of FCS were mullite (3Al_2_O_3_2SiO_2_) and quartz (SiO_2_). The pattern of the FCS-SMS cured at 85 °C (not shown) was identical to that of FCS, suggesting that no crystalline reaction products formed at that temperature.

Two zeolite phases formed after autoclaving FCS-SMS at 175 °C-garronite [(Na_4_Al_4_(SiO_4_)_4_(H_2_O)_7.6_] (patterns overlapping with that of Na-zeolite P) as the major phase and analcime (dehydrated) (Na_7.7_Al_7.7_Si_6.3_O_48_) as a minor one. These two phases are the products of pozzolanic reactions. The pattern of the 250 °C cured sample was very different from the one cured at 175 °C. Analcime, high-temperature zeolite became the major phase, while the intensity of the garronite peaks significantly decreased with some of them disappearing. Likewise, the intensity of mullite and silica peaks reduced significantly, indicating some reactions of FCS. It is reasonable to assume that the formation of analcime as the major crystalline product was due to either the garronite high-temperature transformation or high-temperature SMS reaction with FCS, or both. These data show the susceptibility of FCS to pozzolanic reactions at temperatures ≥175 °C.

**Figure 5:**
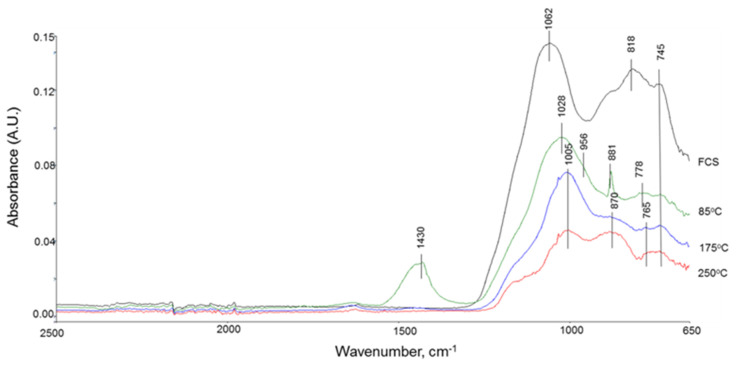
ATR-FTIR spectra of “as-received” FCS and FCS/SMS samples made at hydrothermal temperatures of 85, 175, and 250 °C.

The results of XRD analyses were supported by the FTIR study. Figure 5 gives the ATR-FTIR absorption spectra for the samples analyzed by XRD.

Since the principal crystalline phases of FCS are mullite and silica, the bands at 1062 and 818 cm^−1^ represent the M–O (M: Si or Al) anti-symmetric (*ν_as M–O_*) and symmetric (*ν_s M–O_*) stretching vibrations, respectively, in a Si–O–Al network of mullite and Si–O–Si linkage of silica as tetrahedrally coordinated AlO_4_ and SiO_4_ [39,40]. The 745 cm^−1^ band is Si–O stretching (*ν_s Si–O_*) mode in crystalline and amorphous silica [41,42]. The SMS (Na_2_SiO_3_) aqueous solution with PH > 13 contains SiO_2_(OH)_2_^−2^ anions and 2Na^+^ cations [43]. Thus, although not shown in this figure, the spectrum exhibited the presence of a band at 992 cm^−1^ belonging to the stretching vibration (*ν_Si–O_*) of the Si–O bond in SiO_2_(OH)_2_^−2^, while the water (H_2_O) molecule-related bands can be seen from O–H symmetric stretching vibration (*ν_s O–H_*) at 3348 and 3238 cm^−1^ and H–O–H bending vibration (*δ_H–O–H_*) at 1640 cm^−1^ [44]. When FCS was treated with SMS solution in 85 °C steam, the spectral features drastically altered from that of “as-received” FCS. There were three alterations: (1) the shift of 1062 and 818 cm^−1^ band-related Si- or Al–O peaks to low wavelengths at 1028 and 778 cm^−1^, while the crystalline and amorphous silica-associated band at 745 cm^−1^ remained unchanged; (2) the appearance of sodium carbonate-related CO_3_^2−^ bands at 1430 and 881 cm^−1^ corresponding to C–O anti-symmetric stretching (*ν_as C–O_*) and C–O symmetric (*ν_s C–O_*), respectively, as prominent peaks; and (3) the presence of a shoulder band at 956 cm^−1^ attributed to Si- or Al–OH bending (*δ_Si– or Al–OH_*) mode in silanol (Si–OH) and aluminol (Al–OH) groups [45,46,47] derived from hydroxylation reactions of mullite and silica with SMS. As for the first alteration, such a shift seems to demonstrate the formation of an amorphous Na_2_O-Al_2_O_3_-SiO_2_-H_2_O (N-A-S-H) system brought about by the pozzolanic reactions of aluminosilicate mullite with Na^+^ ions released by SMS, as was shown earlier in the work on SMS-activated FAF/CAC blends [6,48]. Thus, as seen in the absorption spectra, at 85 °C, SMS treatment not only incorporates functional silanol (Si–OH) and aluminol (Al–OH) groups reflected by the shoulder band at 956 cm^−1^ into mullite and quartz within FCS but also forms a new pozzolanic reaction product, amorphous Na-aluminosilicate hydrate compound Na_2_O-Al_2_O_3_-SiO_2_-H_2_O (N-A-S-H), which gives a band at 1028 cm^−1^ as the FCS band at 1062 cm^−1^ shifts to low wavelength.

**Figure 6:**
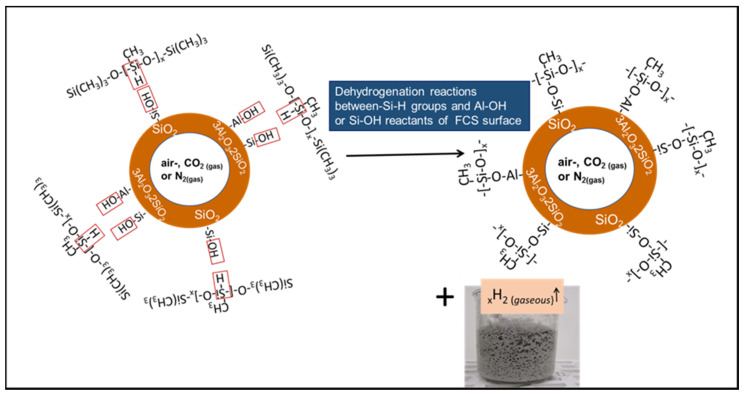
Dehydrogenation reaction between hydroxylated FCS surfaces and Si–H in PMHS at ambient temperature.

We also evaluated FCS surface hydroxylation with the formation of silanol and aluminol groups at room temperature. The FCS/SMS slurry was left for 24 h at room temperature and then dried for 3 days in a vacuum oven at 60 °C. Although not shown in the figure, the spectrum included silanol- and aluminol-related shoulder bands at 935 cm^−1^ coexisting with O–H stretching vibration (*ν_s O–H_*) in Si- and Al–OH at 3421 cm^−1^. Thus, these functional hydroxyl groups formed on FCS surfaces at room temperature. Importantly, the formation of Si–OH and Al–OH groups on FCS surfaces in the presence of SMS accelerated the dehydrogenation reactions between ≡Si–H in PMHS and OH–M (M: Si or Al) in FCS at 85 °C, ≡Si–OH + OH–M–FCS → ≡Si–O–M–FCS + H_2_↑ (Figure 6).

Compared to the spectrum of the 85 °C cured sample, the autoclaving at 175 °C resulted in three noticeable changes in spectral features: (1) a further shift of Si- or Al–O bond-related peaks to lower wavelengths at 1005 cm^−1^ from 1028 cm^−1^; (2) the emergence of a new wide shoulder band at around 870 cm^−1^; and (3) the disappearance of sodium carbonate-related bands. In agreement with the XRD results, the first two changes can be associated with two zeolitic crystalline reaction products, garronite (or Na(zeolite P)) as a major phase at 1005 cm^−1^ and analcime as a minor phase at around 870 cm^−1^. Thus, the hydrothermal treatment at 175 °C promoted the phase transition of amorphous N-A-S-H at 85 °C to crystalline zeolitic compounds. The results of XRD for elevated temperature of 250 °C were also corroborated by the FTIR study. The band of analcime at 870 cm^−1^ became one of the principal peaks indicating analcime becoming the major phase while the peak intensity at 1005 cm^−1^ of garronite (or Na (zeolite P)) considerably decayed.

#### 3.1.2. Reactions between PMHS and SMS

**Figure 7:**
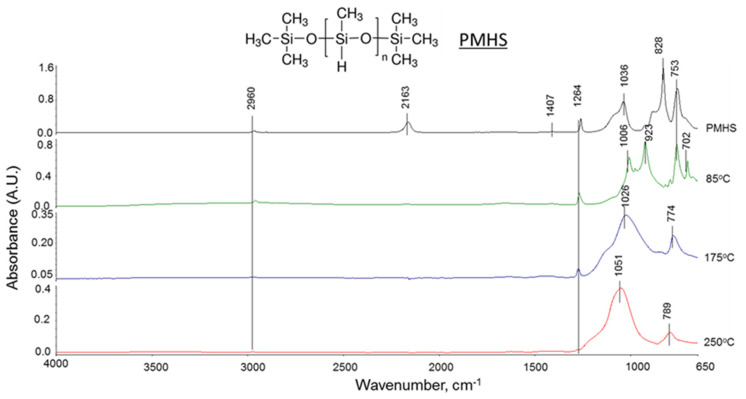
ATR-FTIR spectra of “as-received” PMHS, and PMHS/SMS samples cured at 85, 175, and 250 °C.

Figure 7 gives FTIR spectra of “as-received” PMHS and PMHS/SMS samples cured at 85, 175, and 250 °C. The PMHS’ spectrum included seven bands at 2960, 2163, 1407, 1264, 1036, 828, and 753 cm^−1^. These bands can be assigned as follows [36,37,38,49,50,51]. The 2960 and 1407 cm^−1^ bands are anti-symmetric methyl C–H stretching (*ν_as C__–H_*) and symmetric methyl C–H bending (*δ_s c__–H_*) vibrations in Si–CH_3_ groups, respectively, while the bands at 2163 and 828 cm^−1^ are due to *ν_Si__–H_* and *δ_Si__–H_*, respectively, in the functional Si–H group. Both 1264 and 753 cm^−1^ bands are from the bending mode (*δ_Si__–CH3_*) of the Si–CH_3_ group. The 1036 cm^−1^ band is associated with the Si–O–Si backbone stretching (*ν* *_Si__–O__–Si_*) mode in the siloxane group.

The spectrum of PMHS/SMS blend cured at 85 °C showed two new bands at 923 and 702 cm^−1^, while the Si–H bands at 2163 and 828 cm^−1^ vanished. There was also a striking shift of the Si–O–Si band from 1036 cm^−1^ to a lower wavelength of 1006 cm^−1^. The band of Si–H disappeared because the group reacted with SMS. The two new bands at 923 and 702 cm^−1^ are likely to belong to the reaction products. The potential contributor to these bands is nonbridging Si–O stretching (*ν*_Si__–O_) in the Si–O^−^ Na^+^ complex [52,53,54] and Si–O stretching (*ν_s Si__–O_*) mode in newly precipitated silica. If this interpretation is correct, the following dehydrogenation reactions between Si–H and SMS occurred at 85 °C: 2 ≡Si–H + SiO_2_(OH)_2_^2−^ + 2Na^+^ → 2 ≡Si–O^−^ Na^+^ + 2H_2_↑ + SiO_2 (*precipitation*)_ ↓. On the other hand, a striking shift of the Si–O–Si band may be due to the rupture of Si–O–Si backbone linkages by the formation of the ≡Si–O^−^ Na^+^ complex. Some investigators reported that the bond strength of Si–O–Si adjacent to this complex decreases, thereby resulting in the cleavage of Si–O–Si linkage and depolymerization [55,56]. However, we observed that at ambient temperature in the system of PMHS/SMS/FCS, PMHS preferentially reacts with SMS-activated FCS rather than with SMS alone. So, it is likely that such a complex can form in the case when unreacted PMHS remained at 85 °C.

Interestingly, when the 85 °C-treated sample was autoclaved at 175 °C, the Si–O–Si band reversely shifted to a higher wavelength of 1026 cm^−1^ from 1006 cm^−1^, suggesting the recovery of Si–O–Si linkages cleaved at 85 °C. The cleavage site is susceptible to hydrothermal hydroxylation with the formation of silanol, Si–OH. The self-condensation between silanols, ≡Si–OH + OH–Si≡ → ≡Si–O–Si≡ + H_2_O, results in repolymerization, –[–Si–O–Si–]_n_–, in an alkali hydrothermal environment [57,58,59,60].

There are two other signs of such repolymerization. As is evident from the spectrum at 175 °C, there was no band at 923 cm^−1^ related to the ≡Si–O^−^ Na^+^ complex, while the peak intensity of Si–CH_3_-associated bands at 1264 cm^−1^ and 753 cm^−1^ slightly decreased compared with that at 85 °C. In fact, the ∆A values at the 2163 cm^−1^ band at 85° and 175 °C were 0.017 and 0.014, respectively. These data suggest that the ≡Si–O^−^ Na^+^ complex underwent hydrolytic attacks in the hydrothermal environment at 175 °C, inducing the formation of a silanol (Si–OH) group as the hydrolysate, ≡Si–O^−^ Na^+^ + H_2_O → ≡Si–OH + NaOH. The decreased intensity of Si-CH_3_ bands may be a result of the hydrolysis-led scission of ≡Si–CH_3_ bond in an alkali hydrothermal environment at an elevated temperature at 175 °C [61]. This bond scission substitutes the debonded methyl (CH_3_) groups with hydroxyl (OH) groups, ≡Si–/CH_3_ + H_2_O → ≡SiOH + CH_4*(gaseous)*_↑ with the formation of silanol [62]. Furthermore, the study on the hydrothermal stability of crosslinked liquid silicone rubber after a long time exposure to 100 °C revealed the cleavage of the Si–O–Si backbone as the major degradation mechanism [63], suggesting that the scission of Si–O–Si linkages results not only from alkaline degradation with the formation of the ≡Si–O^−^ Na^+^ complex, but also is likely to be related to the high hydrothermal temperature of 175 °C. Next, all newly formed silanol groups branching from Si–O chains react with each other to produce new ≡Si–O–Si≡ linkages in self-condensation, ≡Si–OH + OH–Si≡ → ≡Si–O–Si≡ + H_2_O [60]. The information above explains the repolymerization taking place at 175 °C. A further advance of repolymerization was observed for samples autoclaved at 250 °C. Among the major changes in the spectral feature of these samples compared to samples cured at 175 °C was a further shift of the Si–O–Si bond to higher wavelength at 1051 cm^−1^ from 1026 cm^−1^, an increase in peak height of the Si–O–Si bond band representing enhanced self-condensation, and very weak Si–CH_3_ bands, implying higher hydrolysis compared with that at 85° and 175 °C.

**Figure 8:**
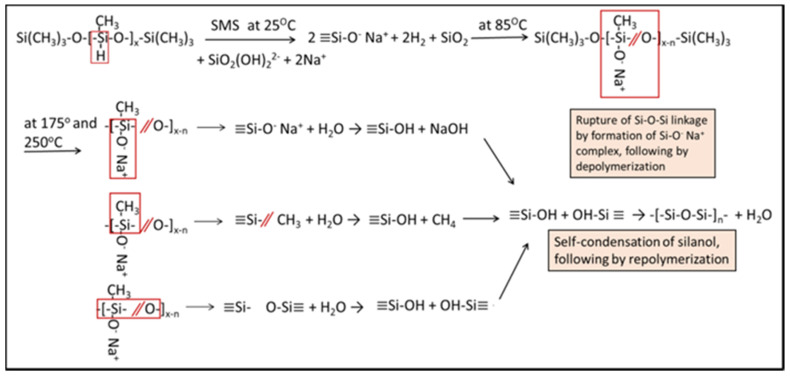
In situ hydroxylation and self-condensation pathways for PMHS/SMS blend at 85, 175, and 250 °C.

All these factors suggest advanced repolymerization through the formation of new oxygen-bridged Si–O–Si linkages at 250 °C. Thus, such repolymerization may lead to in situ hydrothermal transformation of siloxane polymer to highly crosslinked silicone-based polymer creating a three-dimensional structure without carbon as the repolymerization-induced product (RIP). If this postulation is reasonable, silicone RIP may possess a better hydrothermal stability than siloxane. Figure 8 illustrates in situ hydroxylation and self-condensation pathways at 175 and 250 °C of the ruptured Si–O main chain bond among the PMHS/SMS reaction products at 85 °C.

As is evident from a very weak peak of ≡Si–CH_3_ group-related 1264 cm^−1^ band, one concern at 250 °C is a substantial loss of hydrophobic ≡Si–CH_3_ groups, while a certain amount of this group is still present at 175 °C. Although the 250 °C hydrothermal environment aided in achieving the in situ hydroxylation and self-condensation, the loss of this group may reduce the extent of hydrophobicity for 250 °C-autoclaved well cements.

**Figure 9:**
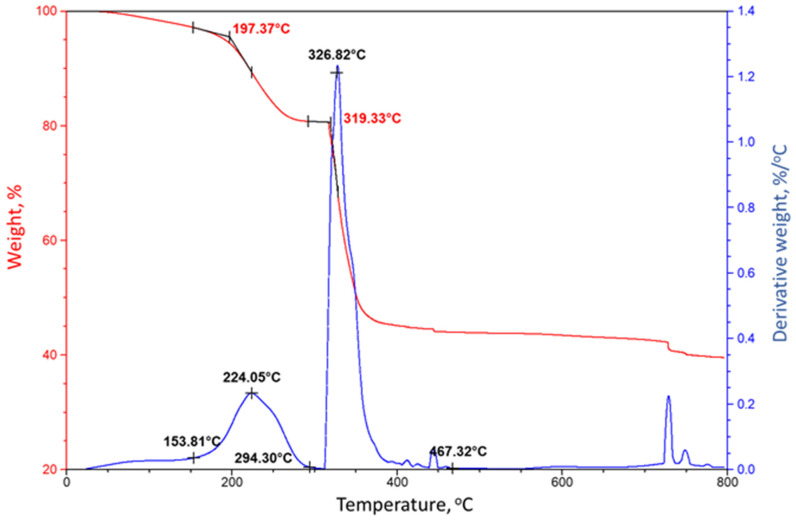
TGA and DTG curves for “as-received” PMHS liquid.

The thermal stability of siloxane at 85 °C and RIP formed by repolymerization at 250 °C was evaluated using thermogravimetric analyzer. Firstly, the thermal stability of “as-received” PMHS was determined (Figure 9). The TGA curve showed two thermal degradation regions: one starting at ≈197 °C and the second one starting at ≈319 °C. The analysis of the differential thermal gravimetric (DTG) curve showed that the first degradation occurred between about 154 and 224 °C. Since the boiling temperature of PMHS is ≈205 °C, this weight loss was due to the volatilization of an organic CH_3_ group in the Si–CH_3_ bond, corresponding to the weight loss of ≈14%. The second region of the major weight loss of ≈36% occurred between ≈319 and 467 °C and was related to the depolymerization of PMHS through Si–O bond scission in Si–O–Si linkages [61,64,65].

**Figure 10:**
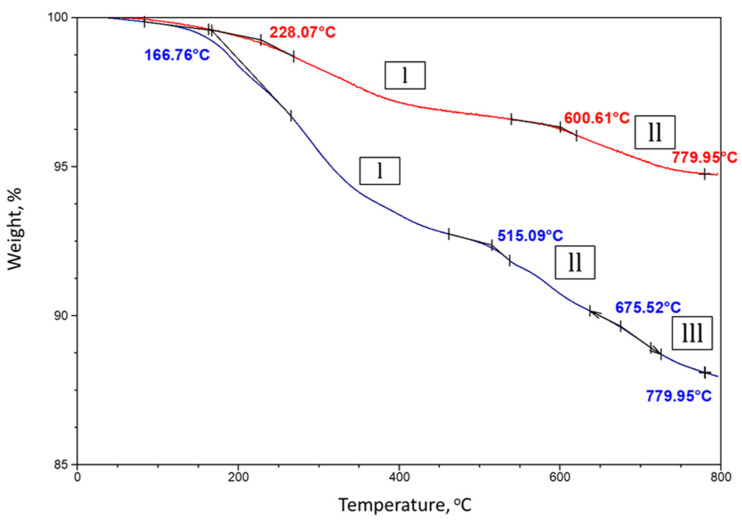
TGA curves of 85 °C-steam cured and 250 °C-autoclaved PMHS/SMS samples.

Figure 10 shows TGA curves of PMHS/SMS samples cured at 85 and 250 °C. The curve for the 85 °C-cured sample showed three decomposition regions up to ≈780 °C. The onset temperatures of these regions were ≈167 °C, ≈515 °C, and ≈676 °C. During the first decomposition stage, 7% of the sample’s weight was lost due to the volatilization of organic CH_3_ groups and depolymerization through multiple scissions of long Si–O–Si backbone chains. Thus, the thermal stability of PMHS with SMS increased to ≈515 °C from ≈319 °C for liquid PMHS. After that, there was a continuous weight loss of 2.7% up to ≈676 °C. Conceivably, the second degradation can be due to the decomposition of –Si–O^−^ Na^+^ -complex, as a reaction product. If so, the reactions between PMHS and SMS at 85 °C offered improved polymer stability. The last degradation region above ≈676 °C can be associated with weight losses from mineral salts or silica or both.

For the 250 °C-autoclaved sample, the first and second degradation onset points shifted to higher temperatures of ≈228° and ≈600 °C. The first stage of thermal degradation weight loss of 2.9% was lower than that for the 85 °C-cured sample and was the result of the depolymerization of some original siloxanes, since most of the CH_3_ groups were eliminated during the 250 °C autoclaving. The second decomposition could be from RIP. If this is the case, the conversion of siloxane polymer → RIP enhances the thermal stability of the solid polymer, increasing the degradation temperature from ≈515 °C for –Si–O^−^ Na^+^ complex forming at 85 to ≈600 °C for RIP that forms at 250 °C. The weight loss of 1.5% at the second decomposition stage was nearly two times lower than for the complex, suggesting that some PMHS may have leaked out during repolymerization at 250 °C.

#### 3.1.3. Reactions between PMHS/FCS/SMS

**Figure 11:**
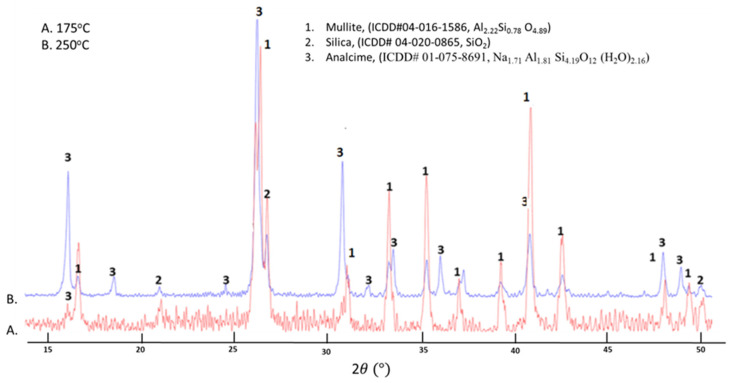
XRD patterns for 175 °C- and 250 °C-autoclaved PMHS/FCS/SMS blend.

This study was done to evaluate the effectiveness of deprotonated PMS to prevent pozzolanic reactions of FCS in the presence of SMS through the formation of the interfacial chemical bond: ≡Si–O–M–FCS (M: Si or Al). Figure 11 provides the XRD patterns and crystalline phases identified from these patterns for samples cured at 175° and 250 °C. At 175 °C, many unreacted FCS were present, as is evident from the strong peak intensities of mullite and silica, while the weak analcime-related peaks demonstrated that the presence of analcime as the reaction product between FCS and SMS was small, if any. Interestingly, there was no garronite (or Na(zeolite P)) phase that was formed in the FCS/SMS system at the same temperature. Since zeolites are reaction products of FCS pozzolanic reactions, this finding strongly verified that the PMHS/FCS interfacial bond was strong enough to impede pozzolanic reactions of FCS with SMS, providing FCS chemical inertness to these reactions at 175 °C. However, temperature increase to 250 °C engendered the loss of the chemical inertness. The peak intensity of analcime increased significantly, while the intensity of mullite and silica peaks decreased at 250 °C, suggesting the breakage of the interfacial chemical bond, ≡Si–O–//–M–FCS, at 250 °C.

**Figure 12:**
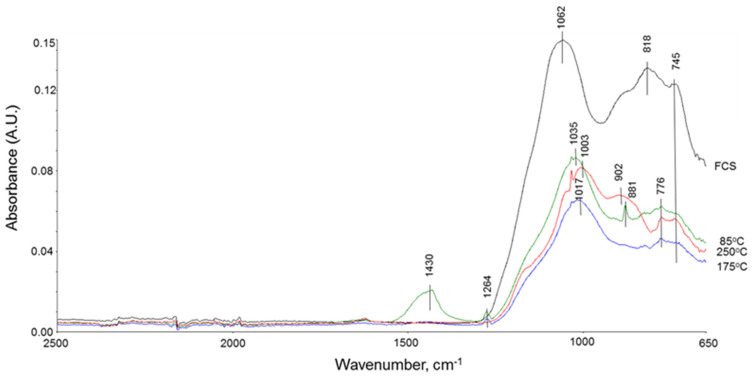
ATR-FTIR spectra for ^“^as-received” FCS, and SMS/PMHS/FCS blends at 85, 175, and 250 °C.

The features of the PMHS, SMS, and FCS blend ATR-FTIR spectra closely resembled those of the FCS/SMS system (Figure 12). Nevertheless, there were two major differences: One was the absence of the analcime-related band in the region between 905 and 850 cm^−1^ for samples cured at 175 °C; the other was the appearance of analcime as a minor phase (the shoulder band at 902 cm^−1^) for the sample cured at 250 °C. Meanwhile, the SiO–M (M: Si or Al) bond-related primary band at 1062 cm^−1^ and secondary band at 818 cm^−1^ in FCS shifted to lower wavelengths at 1035 and 776 cm^−1^ at 85 °C. A further shift of the primary band to 1017 and 1003 cm^−1^ took place at 175° and 250 °C, respectively, while for the secondary band, the shift was the same as at 85 °C. Since XRD data did not show the presence of garronite in samples cured at 175° and 250 °C, all primary bands at 85°, 175°, and 250 °C were attributed to amorphous pozzolanic reaction products: N-A-S-H. Thus, the combined information of XRD and ATR-FTIR studies verified that the reaction products formed in the PMHS/FCS/SMS blend at 175 °C were essentially amorphous N-A-S-H phase. On the other hand, this finding strongly suggested that the unreacted FCSs with PMHS were still present in this system. At 250 °C, the reaction products included amorphous N-A-S-H as the major phase and analcime crystals as the minor phase. Nonetheless, compared with that of PMHS-free FCS/SMS blend, PMHS considerably reduced the extent of FCS pozzolanic reaction that could lead to the degradation of the FCS shell and release of encapsulated gases.

**Figure 13:**
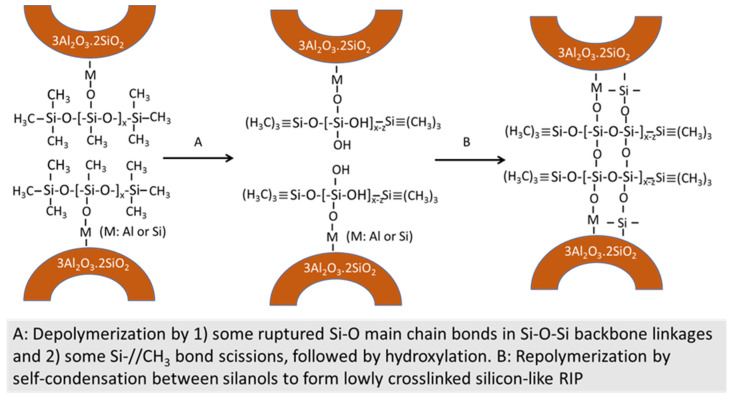
Partial depolymerization and repolymerization pathway of PMHS bonded to the FCS surface at 175 °C.

**Figure 14:**
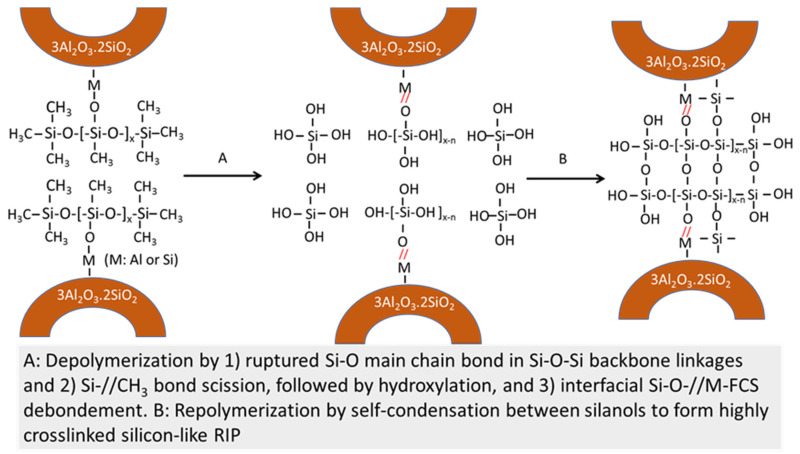
Depolymerization → repolymerization of PMHS debonded from the FCS surfaces at 250 °C.

Based upon the information described above, Figure 13 and Figure 14 illustrate in situ siloxane depolymerization and conversion to crosslinked silicon-like RIPs at 175 and 250 °C in the PMHS/FCS interfacial boundary region.

### 3.2. Cement Slurry Properties

**Figure 15:**
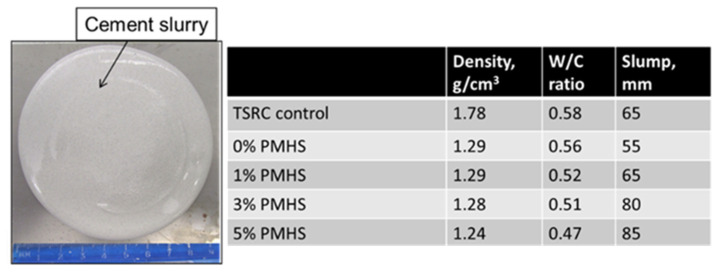
Photograph of cement slurry with 5%-PMHS-treated FCS and a table with slurry properties at different PMHS concentrations used to treat FCS.

Figure 15 shows a photograph of cement slurry with (5% PMHS)-treated FCS and a table that compares the density and slump values of reference and lightweight cement slurries made with W/C ratios (where C is the total CAC and FCS weight), ranging from 0.47 to 0.58. The water was added to the slurry until water bleeding was observed (appearance of water at the surface). The reference cement slurry of TSRC had the density of around 1.78 g/cm^3^ at 0.58 W/C ratio, and 65 mm slump. The density of the lightweight slurry with non-treated FCS (0% PMHS) was 29% lower, 1.29 g/cm^3^, with a W/C ratio of 0.56. The slump decreased from 65 to 55 mm for the lightweight slurry with non-treated PMHS, suggesting the decreased workability of the slurry. Treatment of FCS with PMHS resulted in increased slurry fluidity (increased slump) at lower W/C ratios. The slump was 80 and 85 cm for the PMHS treatment of 3 and 5%, respectively. The changes in the slurry properties for PMHS-treated FCS are probably due to the lubricant effect of silicon–oil-based PMHS. The W/C ratio decreased from 0.56 for non-treated FCS to 0.47 for FCS treated with 5% PMHS. Thus, the lubricating effect of PMHS allowed good slurry flow with decreased water content. The densities of the slurries with PMHS-treated FCS varied between 1.24 and 1.29 g/cm^3^.

### 3.3. Excessive PMHS Amounts in Cement Slurries

**Figure 16:**
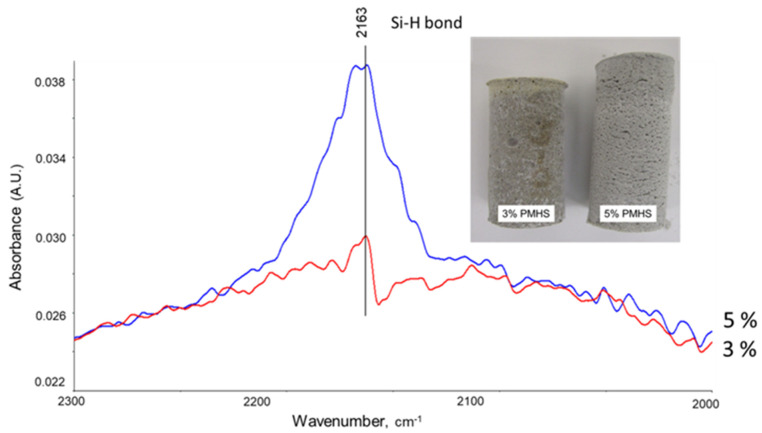
Photograph of cement (CAC/FCS/SMS) volumetric expansion with the 5%-PMHS-treated FCS and comparison of absorbance peak height at 2163 cm^−1^ attributed to unreacted Si-H for 3 and 5% PMHS-treated FCS samples.

If non-reacted PMHS remains in the cement slurry, it may interact with CAC. Samples prepared with FCS treated with 5% PMHS underwent nearly 19% volumetric expansion after 24 h at room temperature, while those with FCS treated with 3% PMHS did not change in size (Figure 16). It is reasonable to assume that the expansion was caused by the reactions of Si–H groups that remained non-reacted after the pre-treatment of FCS. To analyze the PMHS that remains non-reacted during the treatment of FCS, the blend of FCS/SMS was treated with 3 and 5% PMHS and left for 72 h at ambient temperature; then, the Si-H related absorbance peak 2163 cm^−1^ was compared for the two PMHS concentrations (Figure 16). The peak was clearly much higher for the blend with 5% PMHS, confirming the presence of non-reacted Si–H groups at high PMHS concentration.

This means that in the cement system that includes CAC/FCS/SMS, the unreacted Si-H groups of PMHS interact with CAC hydrates of calcium and aluminum. To verify the interactions of CAC with PMHS, a slurry of CAC and SMS without FCS was prepared with 5% PMHS. Shortly after the mixing, an intensive dehydrogenation reaction between CAC hydrates and PMHS in the presence of SMS created an aggressive foaming in the slurry. This dehydrogenation reaction took place between Si–H and Ca^2+^, Al(OH)_4_^−^, and OH^−^ dissociated in a very early period of CAC hydration. The foamed cement slurries were autoclaved at 175° and 250 °C for 24 h, which was followed by XRD and ATR-FTIR analyses to obtain information on the hydrothermal reaction products.

**Figure 17:**
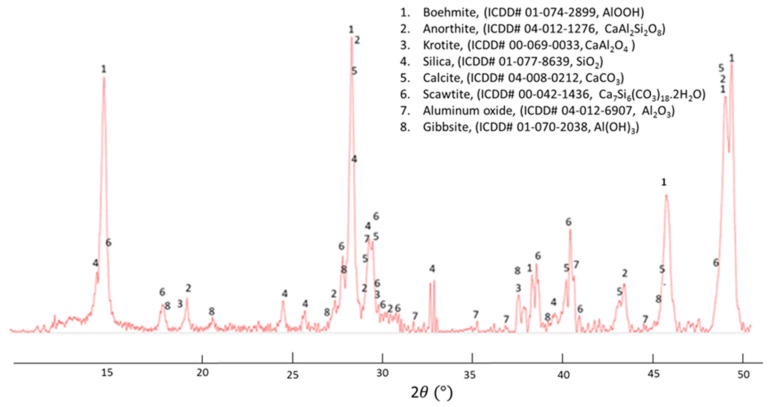
XRD pattern of foam cement made by dehydrogenation reactions between CAC hydrates, PMHS, and SMS, which was followed by autoclaving at 250 °C.

The XRD pattern (Figure 17) revealed multiple crystalline reaction products including boehmite, AlOOH, anorthite, CaAl_2_Si_2_O_8_, calcite, CaCO_3_, scawtite, Ca_7_Si_6_(CO_3_)O_18_·2H_2_O, and gibbsite, Al(OH)_3_, coexisting with unreacted starting materials, krotite, CaAl_2_O_4_, and aluminum oxide, Al_2_O_3_, in CAC, as well as silica, SiO_2_, from SMS. The quantitative data suggested that among the major phases were boehmite (23%), anorthite (19%), and scawtite (14%). Both anorthite and scawtite came from hydrothermal reactions of CAC with silica derived from SMS. (Note that the high-temperature crystalline products of CAC hydration include katoite and boehmite, and the XRD patterns of the hydrated cement can be found in [4].)

**Figure 18:**
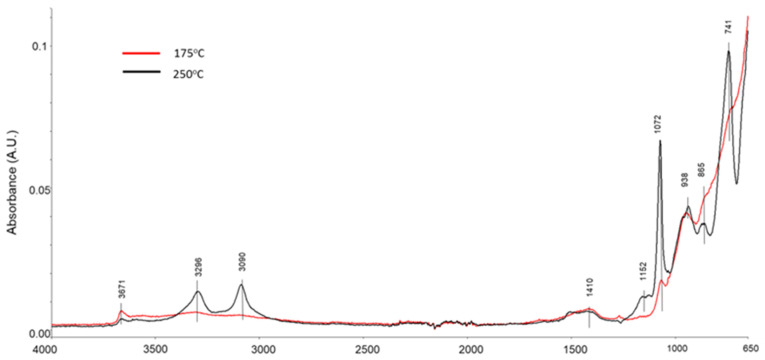
ATR-FTIR spectra of 175 and 250 °C autoclaved samples of CAC/PMHS/SMS.

The ATR-FTIR spectra (Figure 18) of 175 and 250 °C autoclaved CAC/PMHS/SMS blends encompassed nine bands at 3671, 3296, 3090, 1410, 1152, 1072, 938, 865, and 741 cm^−1^. Based on the XRD identification of the major crystalline phases, the boehmite-related bands included those at 3296, 3090, 1152, 1072, and 741 cm^−1^, which were pertinent to *ν_as O–H_*, *ν_s O–H_*, *δ_as Al–O–H_*, *δ_s Al–O–H_*, and *ν_s octahedral AlO6_*, respectively [66,67,68]. The T–O bond (T, Al, or Si) in the aluminosilicate structure of anorthite appears at 938 cm^−1^ [69], while two bands at 1410 and 865 cm^−1^ belong to CO_3_^2−^ in scawtite and calcite. The remaining band at 3671 cm^−1^ may be the *ν _O–H_* in gibbsite. Thus, the band at 938 cm^−1^ is not only assigned to the T–O bond but also may come from *δ _Al–O–H_* in gibbsite [70]. Furthermore, this band also comes from the 2≡Si–O^−^ Ca^2+^ complex as the dehydrogenation reaction product between PMHS and CAC, 2≡Si–H + Ca^2+^ + 2OH^−^ → 2≡Si–O^−^ Ca^2+^ + 2H_2_, rather than the Al complex, 3≡Si–O^−^ Al^3+^, because aluminum forms boehmite as the major phase derived from the hydration of Ca-depleted calcium aluminate cement. The spectrum of the 250 °C autoclaved sample shows a striking increase in peak intensity of all boehmite-related bands compared with those in the spectrum of the 175 °C-autoclaved sample. This suggests that the calcium complex formation is more predominant at 250 °C than at 175 °C.

### 3.4. Microcalorimetric Study of Setting Behavior of CAC Blend with SMS/PMHS-Treated FCS at 85 °C

**Figure 19:**
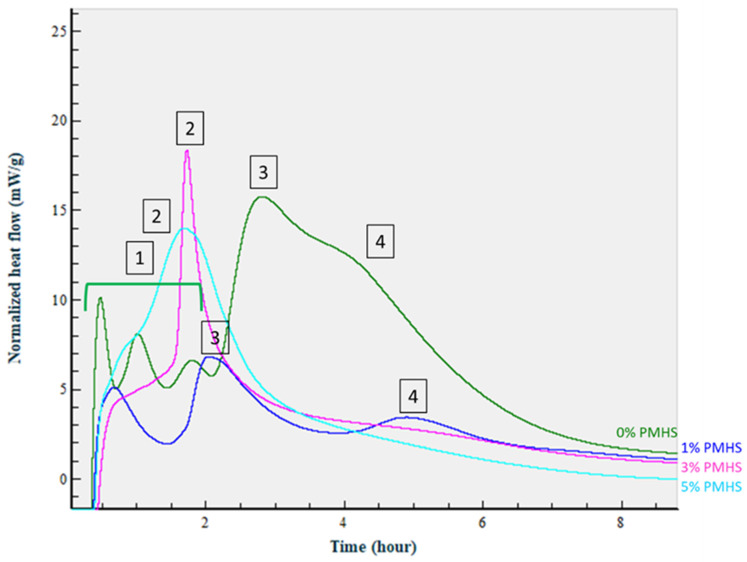
Normalized heat-flow curves at 85 °C isothermal temperature for lightweight cement slurries prepared with 0, 1, 3, and 5% PMHS-treated FCS/SMS and CAC.

Figure 19 shows calorimetric curves for the cement blend with SMS and PMHS-pre-treated FCS along with the curve of the FCS that were pre-treated only with SMS. There are some clear differences in the heat release during slurries hydration for slurries with different amounts of PMHS. Firstly, the initial three peaks (denoted as “1” in the figure) that appeared in the slurry without PMHS vanished from the curves of the slurries with PMHS pre-treatment. Secondly, the major heat release peak for the slurry without PMHS (#3) decreased in intensity and shifted to the earlier time for 1% PMHS. Thirdly, calorimetric curves of the slurries with 3 and 5% PMHS showed a new high heat release peak (#2) that shielded peak #3. Finally, the shoulder (#4) on the 0% PMHS curve transformed into a small peak for 1% PMHS, a long tail for 3% PMHS, and nearly completely disappeared from the heat curve of 5% PMHS slurry. Based on the curves’ comparison, earlier calorimetric studies of CAC/FAF/SMS blends [4], and the studies of interactions between slurries constituents described above, we propose the following interpretation of these results. The first three peaks (#1) of 0% PMHS slurry correspond to the initial CAC dissolution and reactions with FCS surfaces activated by SMS pre-treatment. As the concentration of PMHS increases and FCS surfaces become less reactive, these peaks disappear from the curves. The large peak #3 on 0%-PMHS curve corresponds to the hydration of CAC (integrated heat release for peaks 3 and 4–93 J/g), while the pozzolanic reactions of non-protected FCS produce the right shoulder (#4) on the CAC hydration peak. The curve for 1% PMHS showed an initial cement dissolution peak but no peaks from FCS reactions. The CAC hydration peak shifted to earlier time and decreased in intensity (21 J/g), while the peak of the pozzolanic reactions of 1% PMHS pre-treated FCS (#4) was smaller than that for 0% PMHS slurry and shifted to later hydration time because of the PMHS protection of FCS. As the concentration of PMHS increased to 3 and then 5%, some non-reacted PMHS remained from the pre-treatment of FCS. When mixed with CAC, the dehydrogenation reaction between non-reacted PMHS and CAC produced an additional peak (#2) at early hydration times. In the case of 5%-PMHS, there was a higher concentration of the remaining polymer, so the dehydrogenation reaction started immediately after the mixing before the calorimetric measurements began. As a result, a part of the heat peak was not registered, causing a wider and flatter peak #2 compared with 3% PMHS slurry (63 J/g for 3% PMHS and 53 J/g for 5% PMHS). The dissolution and hydration reactions of CAC (#3 peak in 0 and 1% PMHS) took place continuously during the dehydrogenation. The pozzolanic reactions of FCS were almost or completely eliminated in the 3 and 5% PMHS slurries, respectively (tailing off heat curves). This interpretation of the heat release curves was supported by the monitoring of cement hardening at 85 °C. In cement hardening tests, 20 g of the slurry were poured into 30cc plastic cups, sealed, and exposed to the steam-saturated 85 °C environments. The setting time was monitored by hand mixing the slurries every 2 h. The 0% slurry set after about 8 h, the 1% one set after about 6 h, and the slurries with 3 and 5% PMHS set after 4 h, demonstrating that the slurries set by the end of the intensive heat release. The study shows that the set was accelerated by PMHS pre-treatment of FCS.

### 3.5. Compressive Strength and Toughness

**Figure 20:**
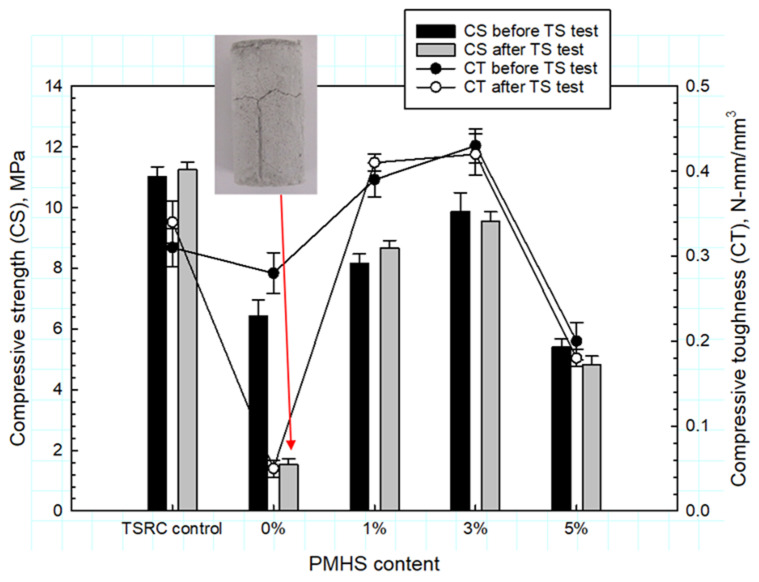
Compressive strength and toughness of 250 °C-autoclaved TSRC control and CAC lightweight cements containing 0, 1, 3, and 5% PMHS-treated FCS before and after the TS test.

Figure 20 shows the compressive strength and toughness before and after the TS test for 250 °C–24 h autoclaved PMHS-treated and untreated lightweight cements. The cement with untreated FCS developed typical volumetric expansion-induced cracks caused by pozzolanic reactions of FCS in cement matrix (the photograph in the figure). This cement failure demonstrates that untreated FCS are very susceptible to pozzolanic reactions during the cyclic 250 °C heat → 25 °C water quenching TS test. These samples lost 76% and 71% of the compressive strength and toughness, respectively, during the test. Both the initial compressive strength and toughness rose with the increase in PMHS content up to 3%. The 3% PMHS treatment offered a strength and toughness increase of 53 and 43%, respectively, up to 9.87 Mpa and 0.43 N-mm/mm^3^, compared with those without PMHS treatment, showing that the treatment not only prevented pozzolanic degradation of FCS but also improved the cement’s mechanical properties. Since a good toughness comes from the proper balance of strength and ductility, the silicone polymer-like RIP formed during depolymerization → repolymerzation of PMHS at 250 °C appears to confer excellent ductility on lightweight cement. With 5% PMHS, the strength and toughness decreased by 45 and 53%, respectively, compared to the 3% PMHS-treated FCS-containing samples because of the foaming of cement matrix due to the excessive unreacted PMHS remaining after the FCS treatment. The foaming created a weak porous matrix. The strength and toughness of 3 and 5% PMHS samples decreased after the TS test; however, the samples’ integrity persisted, highlighting that RIP is able to withstand both thermal and hydrothermal environments at 250 °C. Thus, all the samples with PMHS-treated FCS displayed an excellent TS resistance.

### 3.6. Thermal Conductivity

**Figure 21:**
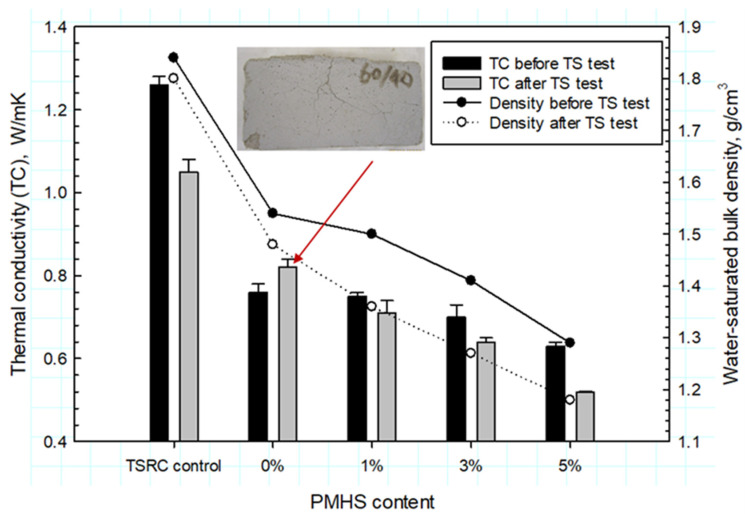
Thermal conductivity and water-saturated bulk density for 250 °C-autoclaved TSRC control, and CAC lightweight cements containing 1, 3, and 5% PMHS-treated and untreated FCSs before and after the TS test.

Figure 21 gives TC and water-saturated bulk density (WSBD) for 250 °C–24 h autoclaved lightweight cements before and after the TS test. Before the TS test, the TC of the control was 1.26 W/mK, and the WSBD was 1.84 g/cm^3^. As expected, the value of TC depended primarily on the value of WSBD; the TC value decreased as the density decreased. The PMHS-free lightweight cement (0%) had 0.76 W/mK TC, 1.54 g/cm^3^ WSBD, and 1.28 g/cm^3^ slurry density, which was a nearly 40% decrease in TC, 16% decrease in WSBD, and 28% lower slurry density than for the control. Importantly, the PMHS treatment further decreased both TC and WSBD values. This decrease was more important for higher PMHS concentrations. Hence, PMHS-treated FCSs offered the improved thermal insulation property of the lightweight cement. There are two principal factors for this: the alkali inertness of FCS surfaces to PR, thereby enabling the preservation of the gaseous thermal insulators encapsulated in the FCS shell and low water absorption and transportation of cement. The contributor to these two factors is likely to be silicone-like RIP. With regard to the alkali inertness of FCS surfaces, as aforementioned in the PMHS/FCS/SMS system, the siloxane oxygen-linked FCS structure, ≡Si–O–M–FCS (M: Si or Al) withstood at 175 °C and protected FCS against PR. However, at 250 °C, the interfacial bonds started breaking, ≡Si–O–//–M–FCS. Thus, the presence of RIP at the surface of FCS was likely helping inhibit the PR of FCS at 250 °C. Furthermore, the lowering of WSBD by low water absorption and transportation is due to a water-proofing effect of RIP. With 5% PMHS, the lowest WSBD of 1.29 g/cm^3^ resulted in the lowest TC of 0.63 W/mK, which was 50% below that of the control. In fact, the free water content of this formulation was 33.8%, which was 18.6% less than that of 0% PMHS cement.

The TC of all the samples decreased after the TS test. The exception was the 0% PMHS sample, which developed PR-induced cracks (see the photograph). The trends for TC and WSBD were similar to those before the TS test; the TC and WSBD values declined with increasing PMHS contents. The reasons for the decrease of TC and WSBD after the TS test may be the improved waterproofing of cement by RIP. The WSBD and free water contents also were lower after the TS test. The free water content for the 0, 1, 3, and 5% PMHS was 42, 26, 25, and 22%, respectively. Consequently, the 5% PMHS sample had the lowest TC of 0.52 W/mK.

### 3.7. Water-Repellent Property

**Figure 22:**
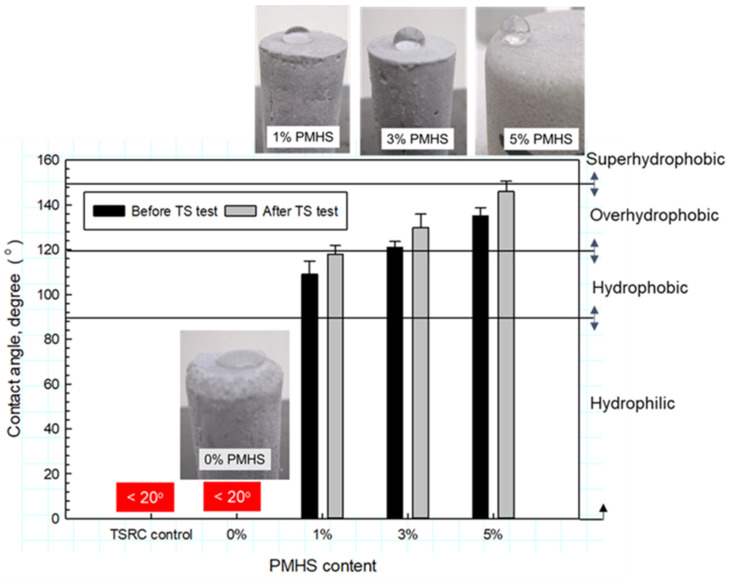
Water contact angles for the control and 250 °C-autoclaved CAC samples containing 1, 3, and 5% PMHS-treated and untreated FCS before and after TS test.

To evaluate the water-repellent property of cement surfaces dried in air for 7 days at ambiance temperature before and after TS test, the contact angle of a water droplet over the cement surface was measured and related to the surface hydrophobicity. Cement was categorized as superhydrophobic for the contact angle >150°, overhydrophobic for the angle range between 150° and 120°, hydrophobic, for the range from 120° to 90°, and hydrophilic for the angle <90° [26]. Figure 22 shows the changes in contact angle as a function of PMHS content for pre- and post-TS test samples along with the photographs of the water droplets on samples after the TS tests. As seen in the photograph, the contact angle of PMHS-free cement (0% PMHS) was less than 20°, the water droplets were spread over the surface, and the cement was hydrophilic with very poor water repellency. By contrast, all samples made with PMHS-treated FCS displayed hydrophobic properties, resulting in the contact angle of >100° for both pre-and post-TS test samples. The upward trend of contact angle value was observed as the PMSH content increased. Furthermore, the contact angle of the post-TS test samples was higher than that of the pre-TS samples, seemingly revealing the improved ability of silicone-like RIP to enhance water repellency during the TS test procedure. For pre- and post-TS test samples, 1% PMHS was classified as hydrophobic, while 3 and 5% PMHS provided an upgraded water repellency as overhydrophobic cements. With 5% PMHS, the contact angle of 132° for pre-TS and 143° for post-TS was 21% higher than those of 1% PMHS. This observation of increased water repellency agrees with the results of TC and wet bulk density measurements, suggesting that the water-proofing property of the silicon-like RIP decreased water absorption and transportation through the cement, allowing the preparation of thermally insulating lightweight cement by incorporating PMHS-treated FCS in CAC at 250 °C.

**Figure 23:**
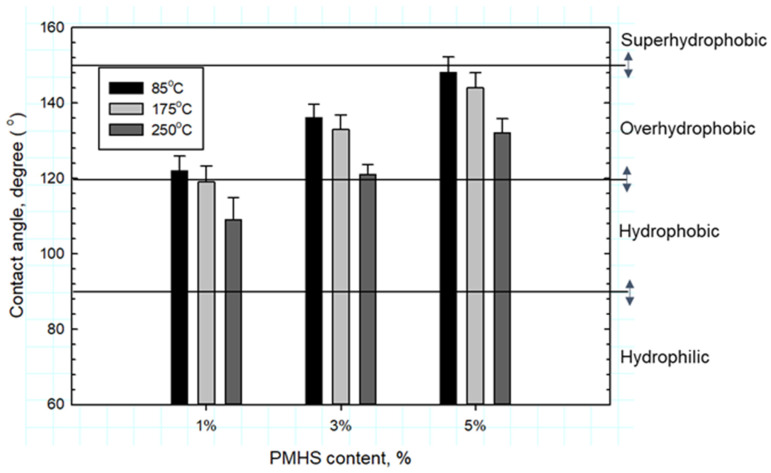
Water contact angle for 85°, 175°, and 250 °C-autoclaved CAC samples containing 1, 3, and 5% PMHS-treated FCS.

As aforementioned in the PMHS/SMS system, alkali hydrolysis-induced bond scission of the Si–CH_3_ group at 250 °C takes place. Since the Si–CH_3_ group provides the hydrophobicity of PMHS, this bond scission may reduce the hydrophobicity of cement. To verify this, the samples with 1, 3, and 5% PMHS-treated FCS were cured in steam at 85 °C and then autoclaved at 175 and 250 °C for 24 h, which was followed by drying for one week in air at ambient temperature for contact angle measurements. Figure 23 shows the changes in contact angle as a function of the hydrothermal temperature. For all samples, the contact angle reduced with increasing temperature. The decrease in contact angle was on average 2.5% for temperature increase between 85 and 175 °C, and it was 8.6% for temperature increase from 175 to 250 °C. Hence, although the silicone-like RIP forms at 250 °C, this high hydrothermal temperature is likely to impair the water repellency of cement due to Si–CH_3_ bond rupture.

### 3.8. XRD Phase Identification

The degradation of FCS shells in alkaline cement environments at high temperatures was the major issue for utilizing FCS as a thermal insulator. The degradation of the shells could be monitored by following the formation of the crystalline PR products of FCS. On the other hand, the crystalline products of CAC hydration provide cement matrix strength. To follow the shells’ stability and the degradation and formation of CAC reactions products, the identification of crystalline reaction products was performed on cements with non-treated FCS and FCS treated with PMHS, which were autoclaved at 250 °C for 24 h.

**Figure 24:**
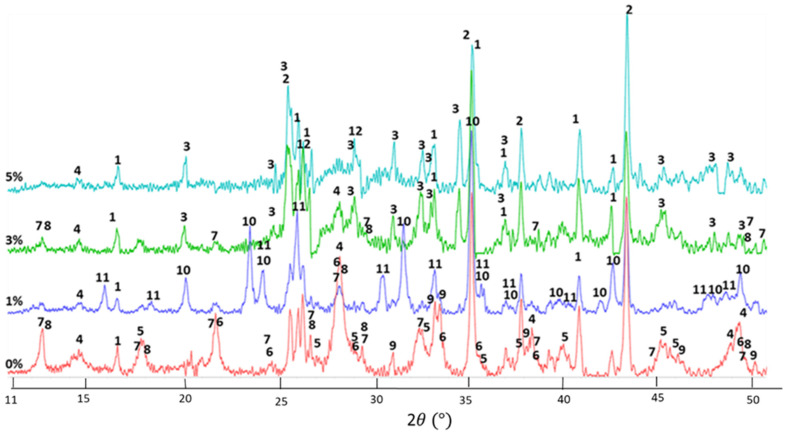
XRD patterns for 250 °C-autoclaved CAC samples containing 1, 3, and 5% PMHS-treated and untreated FCSs: No.1 mullite (ICDD#04-016-1586, Al_2.22_Si_0.78_O_4.89_), No.2 corundum (01-073-1512, Al_2_O_3_), No.3 grossite (01-072-0767, Ca(Al_4_O_7_)), No.4 boehmite (01-073-6509, AlOOH), No.5 katoite (04-014-9841, Ca_3_ Al_2_ (SiO_4_)_1.53_ (OH)_5.88_), No.6 anorthite (01-089-1477, (Ca_0.98_Na_0.02_)(Al_1.98_Si_0.02_)Si_2_O_8_), No.7 gismondine (01-076-8378, Ca_4_(Al_8_Si_8_O_32_ )(H_2_O)_19_), No.8 gobbinsite (01-079-6023, (Na_4.74_Ca_1.23_)(Al_5.65_ Si_10.35_ O_32_ )(H_2_O)_10.9_), No.9 garronite-Na (04-023-2956, Na_3_Al_3_Si_5_ O_16_(H_2_O)_3.6_), No.10 dmisteinbergite (04-011-5220, CaAl_2_Si_2_O_8_), No.11 analcime (04-011-7963, Na_8_Al_8_Si_16_O_48_ (H_2_O)_8_), and No. 12 silica (04-008-8437, SiO_2_).

Figure 24 shows XRD patterns for CAC made with 1, 3, and 5% PMHS-treated and non-treated FCS. A previous study of CAC/FAF/SMS blends showed that the reaction products of CAC cement and SMS released ions (SiO_2_(OH)_2_^2−^ and Na^+^) were garnet series minerals, such as katoite (Ca_3_Al_2_(SiO_4_)_3−x_(OH)_4x_), aluminum oxide hydroxide (boehmite), and feldspar group minerals, such as anorthite and dmisteinbergite (Na_x_Ca_1−x_Al_2−x_Si_2+x_O_8_), while the crystalline products of pozzolanic reactions of FAF were calcium and sodium zeolites and silica [4]. Based on this information and the crystalline phases analysis, it is possible to understand whether PMHS treatment prevents FCS degradation in PR.

The PMHS-free cement encompassed ten crystalline compounds: (1) mullite from unreacted FCS; (2) corundum from CAC; (3) grossite from unreacted CAC; (4) silica derived from the dissolution of SMS and PR of FCS; CAC hydration products (5) boehmite; (6) katoite (garnet series), and (7) anorthite (feldspar group); and zeolites (8) gismondine, (9) gobbinsite, and (10) garronite-Na. Zeolites (8, 9, 10) were FCS reaction products. The strong peaks’ intensities of zeolites suggest that they were well formed in the cement matrix. As mentioned above, while the formation of boehmite, katoite, and anorthite strengthen the matrix, the strong presence of zeolites indicates significant FCS shells degradation in PR.

For the blend with 1% PMHS-treated FCS, the XRD pattern revealed a striking decay of line intensity for these zeolite PR products as well as CAC’s reaction products, including katoite and anorthite. The decrease in the zeolites’ peaks, owing to the partial PMHS protection of the cenospheres’ surface, correlated with the increased intensity of unreacted mullite peaks. The decrease of the CAC reaction products’ peaks suggests that PMHS inhibits reactions with ionic hydrolysates from SMS and/or their crystallization. On the other hand, two new phases, analcime zeolite as the PR product and dmisteinbergite feldspar as CAC’s reaction product, formed. Analcime formation was due to the PR of FCS, indicating that 1% PMHS could not sufficiently protect the surface of the lightweight particles. In contrast, there were three noticeable differences in the pattern of the 3% PMHS-treated FCS blend: (1) an absence of both analcime and dmisteinbergite; (2) further enhancement of mullite-related peak intensity; and (3) the presence of a prominent peak of grossite in CAC. The absence of zeolite products strongly demonstrated that 3% PMHS provided better protection for FCS against PR than 1% PMHS. This is one of the reasons why 3% PMHS offered improved mechanical properties such as compressive strength and toughness. As for the absence of dmisteinbergite and increased intensity of grossite peaks, 3% PMHS seems to restrain the hydration or/and reaction products crystallization of CAC. The weak peaks of two zeolites, FCS’s PR products, gismondine and gobbinsite, were present in the pattern, which means that some FCSs appeared to undergo PR. Compared with 3%, 5% PMHS provided further better protection of FCS against PR, showing no significant signals from any zeolites. Conspicuous peaks of mullite and silica belonging to FCS and grossite in CAC suggested that most of the FCS and CAC as the starting materials remained unreacted.

The only crystalline hydration product of CAC with 5% PMHS-treated FCS samples was boehmite. This finding agrees with the results from the study of interactions between excessive PMHS and CAC at 250 °C; namely, the formation of the 2≡Si–O^−^ Ca^2+^ complex might inhibit the formation of crystalline CAC hydrates. As a result, boehmite preferentially formed from Ca-depleted CAC. The 5% PMHS-induced silicon-like RIPs exerted a better performance in protecting FCS against PR in CAC lightweight cement than 3% PMHS. On the other hand, the inhibition of CAC hydration by PMHS and foaming of the cement matrix due to dehydrogenation reactions between excessive PMHS and CAC would result in a decrease of cement’s mechanical properties. Importantly, RIP played an essential role in strengthening cement with 3 and 5% PMHS-treated FCS.

### 3.9. Microstructural Characterization

**Figure 25:**
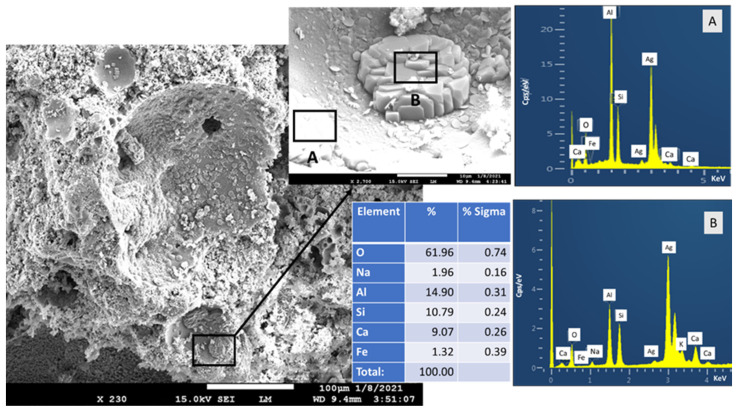
SEM images and EDX elemental composition of CAC blend with untreated FCSs after autoclaving for 24 h at 250 °C.

The microstructural characterization of the samples with untreated FCS and those treated with 3% PMHS was done on samples after 24 h of 250 °C autoclaving. Figure 25 shows an SEM image and EDX elemental composition for the blend with untreated FCS. Most of the FCS suffered from PR. Large- and small-size craters developed because of the FCS shells’ degradation in PR. Correspondingly, when the samples were fractured for the measurements, the fractures propagated through PR-damaged shell structures. As for PR products, the magnified photomicrograph shows two areas with different morphologies and elemental compositions. The area denoted as A represents a relatively smooth surface. For this area, the EDX elemental composition closely resembled that of mullite, Al_2.22_Si_0.78_O_4.89_ (not shown). Therefore, the A area belongs to an unreacted FCS shell. The morphology of the B area was that of pseudo-tetragonal crystal aggregation. The major elemental composition of these crystals was 9.1% Ca, 14.9% Al, and 11.8% Si. Relating this information to the XRD results, these crystals are likely to be gismondine-type zeolite [71,72,73]. These morphological changes indicate that the growth of crystalline zeolite PR products of FCS engendered the volumetric expansion failure of cement in TS tests.

**Figure 26:**
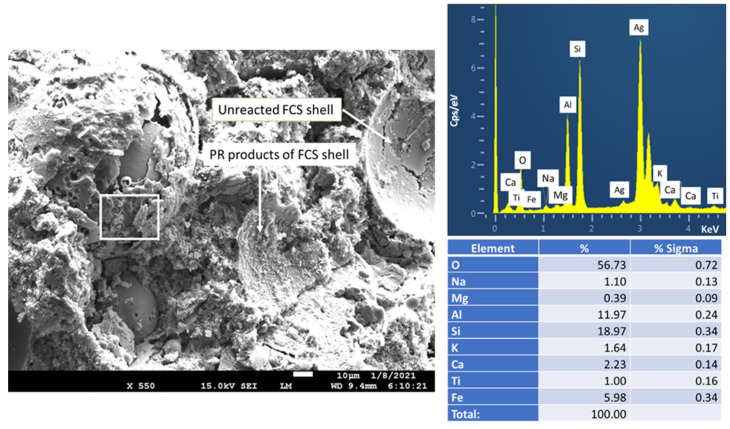
SEM image and EDX elemental composition of CAC blend with 3% PMHS-treated FCS after autoclaving for 24 h at 250 °C.

In contrast, the CAC blend with 3% PMHS-treated FCS (Figure 26) revealed the presence of unreacted FCS shells. The SEM image shows the surface of an entire FCS hallow microsphere covered with amorphous cementitious layer and a FCS shell fractured during the sample preparation with the smooth inner surface. The elemental composition of the amorphous layer on the FCS encompassed three major elements, Si, Al, and O, and five minor elements, Na, K, Ca, Ti, and Fe. Since Ti and Na come from CAC and SMS, respectively, this amorphous-like layer covering the FCS surface may be the reaction product of CAC with the SMS/Na_2_O-CaO-Al_2_O_3_-SiO_2_-H_2_O (N-C-A-S-H) system. On the other hand, as seen in EDX of “as-received” FCS in the starting materials section, the Si, Al, K, Ca, O, and Fe belong to underlying FCS. Additionally, Si and O may also be implicated in the silicon-like RIP. For the FCS cracked open, the smooth inner surface morphology obviously proved that the integrity of the FCS shell remained intact. Thus, two major factors, the formation of amorphous N-C-A-S-H and prevention of PR of FCS by RIP, provided improved mechanical properties of samples treated with PMHS. However, as described in the XRD study, although FCS were treated with 3% PMHS, some PR reaction products of FCS were present, including gismondine (Ca_4_(Al_8_Si_8_O_32_)(H_2_O)_19_) and gobbinsite (Na_4.74_ Ca_1.23_)(Al_5.65_ Si_10.35_ O_32_)(H_2_O)_10.9_). The PR products can be recognized in the image as the rough inner surface texture of one of the shell craters.

**Figure 27:**
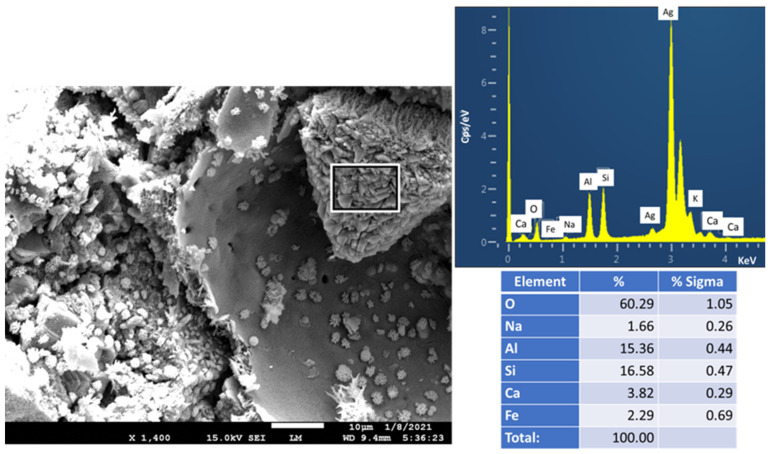
SEM image and EDX elemental composition of CAC blend with 3% PMHS-treated fly ash cenospheres after autoclaving for 24 h at 250 °C.

Figure 27 shows an aggregate with radial morphology of well-crystallized PR product in the FCS crater. Its EDX elemental composition included 15.4% Al, 16.6% Si, 60% O, and 3.8% Ca as major elements and the minor presence of Na and Fe. The elemental percentage ratios of Ca/O, Al/O, and Si/O were 0.06, 0.26, and 0.27, respectively. In contrast, the elemental ratios in molecular formula, Ca_4_(Al_8_Si_8_O_32_)(H_2_O)_19_, of gismondine, obtained from XRD were 0.08 Ca/O, 0.16 Al/O, and 0.16 Si/O. Although Al/O and Si/O ratios of gismondine identified by XRD are 0.1 and 0.11 lower than those obtained by EDX, it is possible to assume that this radial-like crystal aggregate is gismondine, which formed as a PR product of mullite in FCS with Ca^2+^ and 2OH^−^ liberated from the hydrolysis of CAC [71].

**Figure 28:**
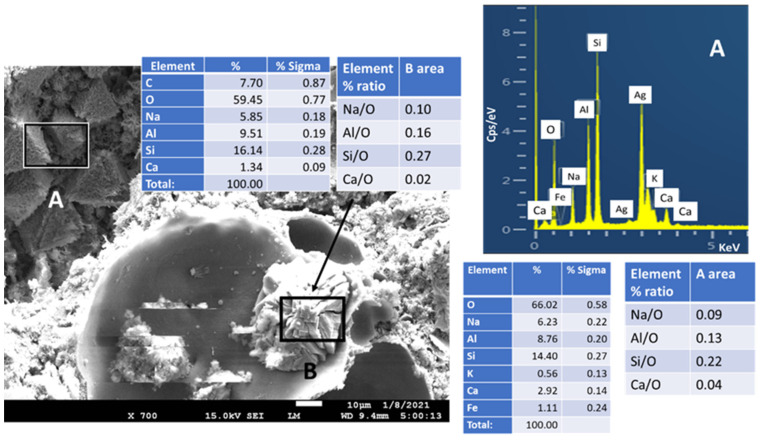
SEM image and EDX elemental composition of CAC blend with 3% PMHS-treated FCSs after autoclaving for 24 h at 250 °C.

Gobbinsite could be identified by its orthorhombic crystal morphology [74,75] (Figure 28). EDX major elemental composition included 6.23% Na, 2.92% Ca, 8.76% Al, 14.4% Si, and 66.02% O, corresponding to the elemental percentage ratios of 0.09 Na/O, 0.04 Ca/O, 0.13 Al/O, and 0.22 Si/O. These elemental ratios closely resembled ratios in the molecular formula, (Na_4_._74_Ca_1.23_)(Al_5.65_ Si_10.35_)O_32_)(H_2_O)_10.9_, of gobbinsite detected in the XRD study; namely, Na/O, Ca/O, Al/O, and Si/O elemental ratios of 0.11, 0.03, 0.13, and 0.24 respectively (area B). In addition, gobbinsite crystals can be seen in area A with similar elemental ratios.

**Figure 29:**
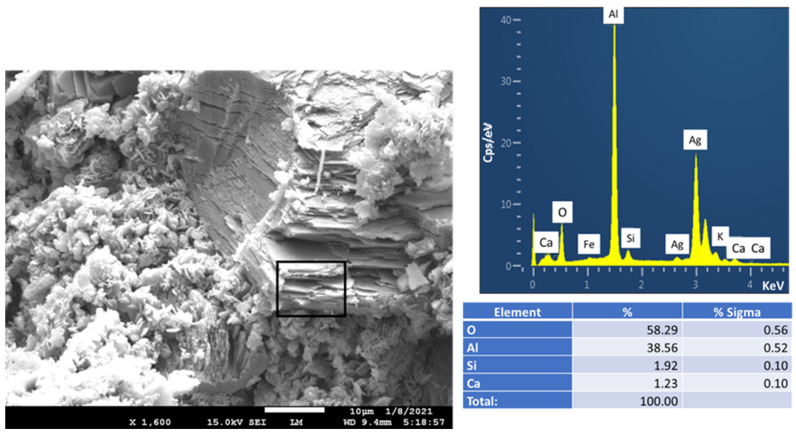
SEM image and EDX elemental composition of CAC blend with 3% PMHS-treated FCSs after autoclaving for 24 h at 250 °C.

Furthermore, XRD study also identified boehmite crystals derived from CAC after calcium reactions with PMHS. Figure 29 shows the plate-like well-formed crystals. EDX elemental composition for this area consisted of two predominant elements, 38.6% Al and 58.3% O, attributed to the boehmite, AlOOH.

## 4. Conclusions

FCS were used as part of the thermal-shock-resistant lightweight cement formulation for underground energy-storage and energy-recovery wells. To minimize the PR of FCS shells constituted of mullite and silica in alkaline environments at hydrothermal temperatures ranging from 85 to 250 °C, the chemical preparation of the shells’ surfaces was performed by blending FCS with SMS and PMHS. This treatment resulted in chemical bonding of FCS with deprotonated PMHS (PMS), minimizing the susceptibility of FCS to PR at up to 175 °C. The elevated temperature of 250 °C brought about a partial breakage of the FCS–PMS interfacial chemical bond.

PMHS molecular structure alterations under high temperatures increased the thermal stability of the FCS protective layer. The presence of original hydrophobic Si–CH_3_ groups within PMS conferred an excellent water-repellent property on lightweight cement at 175 °C. After 250 °C autoclaving, all samples with PMHS-treated FCS were hydrophobic, showing a water droplet contact angle of >100° both before and after the TS test. The contact angle increased with an increasing content of PMHS. Samples with 3 and 5% PMHS were overhydrophobic.

PMHS-treated FCS aggregates were very compatible with CAC, and furthermore, a great lubricating property of PMHS improved the fluidity of cement slurries with low densities, ranging from 1.24 to 1.29 g/cm^3^ and W/C ratios of 0.51–0.47.

The chemical tailoring of the FCS surface by PMHS not only alleviated PR of FCS but also improved mechanical strengths of lightweight cements. In particular, the toughness of 1 and 3% PMHS-treated samples was 26 and 30% higher than that of regular density TSRC control. Although the strength and toughness of 3 and 5% PMHS samples somewhat decreased after the TS tests, their integrity as TS-resistant well cement remained intact.

The incorporation of PMHS declined both TC and WSBD values compared with those of the PMHS-free lightweight cement, strongly demonstrating that PMHS-treated FCSs improved the thermal insulation property of the lightweight cement. Two principal factors were responsible for this: the inertness of PMHS-treated FCS surfaces to PR and the low water absorption and transportation through the hydrophobic cement even under water-saturated conditions of underground wells. With 5% PMHS, the lowest WSBD of 1.29 g/cm^3^ in this test corresponded to the lowest TC of 0.63 W/mK, which was 50% lower than that of the TSRC control. After the TS test, all samples with PMHS-treated FCS further decreased TC; e.g., TC after the TS for 5% PMHS was 0.52 W/mK.

This technology should enable a decrease of energy losses during energy storage and recovery from geothermal wells while providing adequate well integrity.

## 5. Patents

IP2020-012-01, U.S. Provisional Patent Application Serial # 63/087,489 and Title “Super-hydrophobic, thermally insulating, thermal-shocks resistant well cement composites for completion of geothermal wells at hydrothermal temperatures of up to 300 °C”, filed 10 May 2020.

## Figures and Tables

**Table 1:** Oxide compositions of #80 CAC and Class F fly ash.

	Oxide Compositions, wt.%						
	AI2O3	CaO	SiO_2_	Fe_2_O_3_	Na_2_O	K_2_O	TiO_2_
#80 CAC	75.2	24.7	-	0.1	-	-	-
Class F fly ash	35.0	2.7	50.1	7.1	0.30	3.1	1.6

## Data Availability

The data presented in this study are available on request from the corresponding author.

## Abbreviations

CAC	calcium aluminate cement
FAF	fly ash, type F
FCS	fly ash cenospheres
PMHS	polymethylhydrosiloxane
PMS	polymethylsiloxane
PR	pozzolanic reactions
RIP	repolymerization-induced product
SMS	sodium metasilicate
TC	thermal conductivity
TS	thermal shock
TSRC	thermal shock-resistant cement
W/C	water-to-cement ratio
WSBD	water-saturated bulk density

## References

[1] Radenti G., Ghiringhelli G. (1972). Cementing materials for geothermal wells. Geothermics.

[2] Berra M., Fabbri F., Facoetti M., Noris A., Pezzuoli M., Ricciardulli R., Romano G., Tarquini B. (1988). Behaviour of a cementing hydraulic binder under severe geothermal conditions. Geothermics.

[3] Won J., Choi H.-J., Lee H., Choi H. (2016). Numerical investigation on the effect of cementing properties on the thermal and mechanical stability of geothermal wells. Energies.

[4] Sugama T., Pyatina T. (2018). Alkali-Activated Cement Composites for High Temperature Geothermal Wells.

[5] Pyatina T., Sugama T., Zaliznyak T. (2017). Durability of bond between high-temperature cement composites and carbon steel. Proceedings of the Transactions—Geothermal Resources Council.

[6] Sugama T., Pyatina T. (2019). Self-healing, re-adhering, and carbon-steel corrosion mitigating properties of fly ash-containing calcium aluminum phosphate cement composite at 300 °C hydrothermal temperature. Cem. Concr. Compos..

[7] Yu H., Li Q., Sun F. (2019). Numerical simulation of CO2 circulating in a retrofitted geothermal well. J. Pet. Sci. Eng..

[8] Mas A., Guisseau D., Mas P.P., Baufort D., Genter A., Sanjuan B., Girard J.P. (2006). Clay minerals related to the hydrothermal activity of the Bouillante geothermal field (Guadeloupe). J. Volcanol. Geotherm. Res..

[9] Meyer L.L. (2013). Thermophysical Properties of Wisconsin Rocks for Application in Geothermal Energy. Master’s Thesis.

[10] Beziat A., Dardaine M., Gabis V. (1988). Effect of compaction pressure and water content on the thermal conductivity of some natural clays. Clays Clay Miner..

[11] Wang K.S., Tseng C.J., Chiou I.J., Shih M.H. (2005). The thermal conductivity mechanism of sewage sludge ash lightweight materials. Cem. Concr. Res..

[12] Chávez-Valdez A., Arizmendi-Morquecho A., Vargas G., Almanza J.M., Alvarez-Quintana J. (2011). Ultra-low thermal conductivity thermal barrier coatings from recycled fly-ash cenospheres. Acta Mater..

[13] Jung J.S., Park H.C., Stevens R. (2001). Mullite ceramics derived from coal fly ash. J. Mater. Sci. Lett..

[14] Sokol E.V., Maksimova N.V., Volkova N.I., Nigmatulina E.N., Frenkel A.E. (2000). Hollow silicate microspheres from fly ashes of the Chelyabinsk brown coals (South Urals, Russia). Fuel Process. Technol..

[15] Fomenko E., Anshits N., Pankova M. Fly Ash Cenospheres: Composition, Morphology, Structure, and Helium Permeability. Proceedings of the World of Coal Ash Conference.

[16] Yoriya S., Intana T., Tepsri P. (2019). Separation of cenospheres from lignite fly ash using acetone-water mixture. Appl. Sci..

[17] Ignaszak Z., Baranowski A., Hycnar J., Zak M. (1990). Heat-Insulating, High-Temperature Materials on Cenosphere Base. Insulation Materials, Testing and Applicationsl.

[18] Ranibar N., Kuenzel C. (2017). Cenospheres: A review. Fuel.

[19] Lilkov V., Djabarov N., Bechev G., Kolev K. (1999). Properties and hydration products of lightweight and expansive cements Part I: Physical and mechanical properties. Cem. Concr. Res..

[20] Blanco F., Garcia P., Mateos P., Ayala J. (2000). Characteristics and properties of lightweight concrete manufactured with cenospheres. Cem. Concr. Res..

[21] McBride S.P., Shukla A., Bose A. (2002). Processing and characterization of a lightweight concrete using cenospheres. J. Mater. Sci..

[22] Huang X., Ranade R., Zhang Q., Ni W., Li V.C. (2013). Mechanical and thermal properties of green lightweight engineered cementitious composites. Constr. Build. Mater..

[23] Wang M.-R., Jia D.-C., He P.-G., Zhou Y. (2011). Microstructural and mechanical characterization of annealed tungsten. Ceram. Int..

[24] Wang J.Y., Zhang M.H., Li W., Chia K.S., Liew R.J.Y. (2012). Stability of cenospheres in lightweight cement composites in terms of alkali-silica reaction. Cem. Concr. Res..

[25] Smith J.C.B. (1960). Analysis of organo-silicon compounds, with special reference to silanes and siloxanes. A review. Analyst.

[26] Muzenski S., Flores-Vivian I., Sobolev K. (2015). Durability of superhydrophobic engineered cementitious composites. Constr. Build. Mater..

[27] Lin W., Huang Y., Li J., Liu Z., Yang W., Li R., Chen H., Zhang X. (2018). Preparation of highly hydrophobic and anti-fouling wood using poly(methylhydrogen)siloxane. Cellulose.

[28] Hou P., Li R., Li H., Xie N., Cheng X., Singh L.P. (2018). The use of hydrophobicity and pozzolanic reactivity of the PMHS/nanosilica hybrid composites on the water absorption of cement mortar. J. Therm. Anal. Calorim..

[29] Dong B., Wang F., Abadikhah H., Hao L., Xu X., Khan S.A., Wang G., Agathopoulos S. (2019). Simple fabrication of concrete with remarkable self-cleaning ability.pdf. Appl. Mater. Interfaces.

[30] Urhan S. (1987). Alkali silica and pozzolanic reactions in concrete. Part 1: Interpretation of published results and an hypothesis concerning the mechanism. Cem. Concr. Res..

[31] Mertens G., Snellings R., Van Balen K., Bicer-Simsir B., Verlooy P., Elsen J. (2009). Pozzolanic reactions of common natural zeolites with lime and parameters affecting their reactivity. Cem. Concr. Res..

[32] Fernandez R., Martirena F., Scrivener K.L. (2011). The origin of the pozzolanic activity of calcined clay minerals: A comparison between kaolinite, illite and montmorillonite. Cem. Concr. Res..

[33] Pang B., Zhang Y., Liu G., She W. (2018). Interface Properties of Nanosilica-Modified Waterborne Epoxy Cement Repairing System. ACS Appl. Mater. Interfaces.

[34] Wu W., Chen H.L. (2014). Preparation of cyclohexanone/water Pickering emulsion together with modification of silica particles in the presence of PMHS by one pot method. Colloids Surfaces A.

[35] Qiao B., Wang T.J. (2015). High density silanization of nano-silica particles using gaminopropyltriethoxysilane (APTES). Appl. Suface Sci. China.

[36] Assresahegn B.D., Belanger D. (2017). Synthesis of binder-like molecules covalently linked to silicon nanoparticles and application as anode material for lithium-ion batteries without the use of electrolyte additives. J. Power Sources.

[37] Bai H.W., Wen G., Huang X.X., Han Z.X., Zhong B., Hu Z.X., Zhang X.D. (2011). Synthesis and structural characterization of SiBOC ceramic fibers derived from single-source polyborosiloxane. J. Eur. Ceram. Soc..

[38] Matsumura S., Hlil A.R., Lepiller C., Gaudet J., Guay D., Shi Z., Holdcroft S., Hay A.S. (2008). Stability and Utility of Pyridyl Disulfide Functionality in RAFT and Conventional Radical Polymerizations. J. Polym. Sci. Part A Polym. Chem..

[39] Bertoluzza A., Fagnano C., Morelli M., Gottardi V., Guglielmi M. (1982). Raman and infrared spectra on silica gel evolving toward glass. J. Non. Cryst. Solids.

[40] Padmaja P., Anilkumar G.M., Mukundan P., Aruldhas G., Warrier K.G.K. (2001). Characterisation of stoichiometric sol-gel mullite by fourier transform infrared spectroscopy. Int. J. Inorg. Mater..

[41] Saikia B.J., Parthasarathy G., Sarmah N.C. (2008). Fourier transform infrared spectroscopie etimation of crystallinity in SiO2 based rocks. Bull. Mater. Sci..

[42] Guan W., Ji F., Fang Z., Fang D., Cheng Y., Yan P., Chen Q. (2014). Low hydrothermal temperature synthesis of porous calcium silicate hydrate with enhanced reactivity SiO2. Ceram. Int..

[43] Yang X., Roonasi P., Holmgren A. (2008). A study of sodium silicate in aqueous solution and sorbed by synthetic magnetite using in situ ATR-FTIR spectroscopy. J. Colloid Interface Sci..

[44] Efimov A.M., Pogareva V.G., Shashkin A.V. (2003). Water-related bands in the IR absorption spectra of silicate glasses. J. Non. Cryst. Solids.

[45] Sun W., Liu W., Hu Y. (2008). FTIR analysis of adsorption of poly diallyl-dimethyl-ammonium chloride on kaolinite. J. Cent. South. Univ. Technol..

[46] Groza A., Surmeian A. (2015). Characterization of the oxides present in a polydimethylsiloxane layer obtained by polymerisation of its liquid precursor in corona discharge. J. Nanomater..

[47] Nagappan S., Ha C.-S. (2014). Effect of Sodium Hydroxide on the Fast Synthesis of Superhydro-phobic Powder from Polymethylhydrosiloxane. J. Coat. Sci. Technol..

[48] Pyatina T., Sugama T. (2014). Set controlling additive for thermal-shock-resistant cement. Trans. Geotherm. Resour. Counc..

[49] Stafie N., Stamatialis D.F., Wessling M. (2005). Effect of PDMAS cross-linking degree on the permeation perforamnce of PAN-PDMS.pdf. Sep. Purif. Technol..

[50] Wang Y., Ramos I., Santiago-Aviles J.J. (2007). Synthesis of ultra-fine porous tin oxide fibres and its process characterization. Nanotechnology.

[51] Johnson L.M., Gao L., Shields C.W., Smith M., Efimenko K., Cushing K., Genzer J., López G.P. (2013). Elastomeric microparticles for acoustic mediated bioseparations. J. Nanobiotechnology.

[52] Uchino T., Sakka T., Hotta K., Iwasaki M. (1989). Attenuated Total Reflectance Fourier-Transform Infrared Spectra of a Hydrated Sodium Silicate Glass. J. Am. Ceram. Soc..

[53] Roy B.N. (1990). Infrared Spectroscopy of Lead and Alkaline-Earth Aluminosilicate Glasses. J. Am. Ceram. Soc..

[54] Allahdin O., Wartel M., Tricot G., Revel B., Boughriet A. (2016). Hydroxylation and dealumination of a metakaolinite-rich brick underacid conditions, and their influences on metal adsorption: One- andtwo-dimensional (1H,27Al,23Na,29Si) MAS NMR, and FTIR studies. Microporous Mesoporous Mater..

[55] Brady P., Walther J. (1989). Controls on silicate dissolution rates in neutral and basic pH solutions at 25 °C. Geochim. Cosmochim. Acta.

[56] Rees C.A., Provis J.L., Lukey G.C., van Deventer J.S.J. (2007). In Situ ATR-FTIR Study of the Early Stages of Fly Ash GeopolymerGel Formation. Langmuir.

[57] Ducom G., Laubie B., Ohannessian A., Germain P., Chatain V. (2013). Hydrolysis of polydimethylsiloxane fluids in controlled aqueous solutions. Water Sci. Technol..

[58] Purkayastha A., Baruah J.B. (2004). Synthetic methodologies in siloxanes. Appl. Organomet. Chem..

[59] Varaprath S., Stutts D.H., Kozerski G.E. (2006). A primer on the analytical aspects of silicones at trace levels-challenges and artifacts—A review. Silicon Chem..

[60] Chainet F., Le Meur L., Lienemann C.P., Ponthus J., Courtiade M., Donard O.F.X. (2013). Characterization of silicon species issued from PDMS degradation under thermal cracking of hydrocarbons: Part 1—Gas samples analysis by gas chromatography-time of flight mass spectrometry. Fuel.

[61] Camino G., Lomakin S.M., Lazzari M. (2001). Polymethyl silozane thermal degradation: Part I. Kinetic aspects. Polymer.

[62] Singh U.B., Gupta S.C., Flerchinger G.N., Moncrief J.F., Lehmann R.G., Fendinger N.J., Traina S.J., Logan T.J. (2000). Modeling polydimethylsiloxane degradation based on soil water content. Environ. Sci. Technol..

[63] Ghanbari-Siahkali A., Mitra S., Kingshott P., Almdal K., Bloch C., Rehmeier H.K. (2005). Investigation of the hydrothermal stability of cross-linked liquid silicone rubber (LSR). Polym. Degrad. Stab..

[64] Sediri F., Gharbi N. (2005). Room Temperature Synthesis and Characterization of Hybrid Materials of Polymethylhydrosiloxane Modified by Hydroxide Organic Compounds. J. Sol.-Gel Sci. Technol..

[65] Nagappan S., Jo N.J., Lee W.K., Ha C.S. (2017). Thermally stable superhydrophobic polymethylhydrosiloxane nanohybrids with liquid marble-like structure. Macromol. Res..

[66] Hsiang H.I., Liang M.T., Huang H.C., Yen F.S. (2007). Preparation of superhydrophobic boehmite and anatase. Mater. Res. Bull..

[67] Boumaza A., Favaro L., Lédion J., Sattonnay G., Brubach J.B., Berthet P., Huntz A.M., Roy P., Tétot R. (2009). Transition alumina phases induced by heat treatment of boehmite: An X-ray diffraction and infrared spectroscopy study. J. Solid State Chem..

[68] Rajaram K., Kim J. (2017). Surfactant assisted fabrication of different nanostructures of boehmite by hydrothermal process. Int. J. Appl. Eng. Res..

[69] Fernández-Jiménez A., Vázquez T., Palomo A. (2011). Effect of sodium silicate on calcium aluminate cement hydration in highly alkaline media: A microstructural characterization. J. Am. Ceram. Soc..

[70] López A.H., Calvo J.L.G., Olmo J.G., Petit S., Alonso M.C. (2008). Microstructural evolution of calcium aluminate cements hydration with silica fume and fly ash additions by scanning electron microscopy, and mid and near-infrared spectroscopy. J. Am. Ceram. Soc..

[71] Bargar K.E., Oscarson R.L. (1997). Oregon Geology.

[72] Ghobarkar H., Schaf O. (1999). Synthesis of gismondine-type zeolites. Mater. Res. Bull..

[73] Okoronkwo M.U., Mondal S.K., Wang B., Ma H., Kumar A. (2021). Formation and stability of gismondine-type zeolite in cementitious systems. J. Am. Ceram. Soc..

[74] Artioli G., Foy H. (1994). Gobbinsite from Magheramorne Quarry, Northern Ireland. Mineral. Mag..

[75] Armbruster T., Gunter M.E. (2001). Crystal Structures of Natural Zeolites. Rev. Mineral. Geochemistry.

